# The RNA Helicases AtMTR4 and HEN2 Target Specific Subsets of Nuclear Transcripts for Degradation by the Nuclear Exosome in *Arabidopsis thaliana*

**DOI:** 10.1371/journal.pgen.1004564

**Published:** 2014-08-21

**Authors:** Heike Lange, Hélène Zuber, François M. Sement, Johana Chicher, Lauriane Kuhn, Philippe Hammann, Véronique Brunaud, Caroline Bérard, Nathalie Bouteiller, Sandrine Balzergue, Sébastien Aubourg, Marie-Laure Martin-Magniette, Hervé Vaucheret, Dominique Gagliardi

**Affiliations:** 1 Institut de Biologie Moléculaire des Plantes, Centre National de la Recherche Scientifique, UPR 2357, Université de Strasbourg, Strasbourg, France; 2 Platforme Protéomique Strasbourg-Esplanade, Centre National de la Recherche Scientifique, FRC 1589, Université de Strasbourg, Strasbourg, France; 3 Unité de Recherche en Génomique Végétale (URGV), UMR INRA 1165, Université d'Evry Val d'Essonne, Saclay Plant Sciences, ERL CNRS 8196, Evry, France; 4 UMR AgroParisTech-INRA MIA 518, Paris, France; 5 Institut Jean-Pierre Bourgin, UMR1318 INRA-AgroParisTech, Versailles, France; University of California Riverside, United States of America

## Abstract

The RNA exosome is the major 3′-5′ RNA degradation machine of eukaryotic cells and participates in processing, surveillance and turnover of both nuclear and cytoplasmic RNA. In both yeast and human, all nuclear functions of the exosome require the RNA helicase MTR4. We show that the *Arabidopsis* core exosome can associate with two related RNA helicases, AtMTR4 and HEN2. Reciprocal co-immunoprecipitation shows that each of the RNA helicases co-purifies with the exosome core complex and with distinct sets of specific proteins. While AtMTR4 is a predominantly nucleolar protein, HEN2 is located in the nucleoplasm and appears to be excluded from nucleoli. We have previously shown that the major role of AtMTR4 is the degradation of rRNA precursors and rRNA maturation by-products. Here, we demonstrate that HEN2 is involved in the degradation of a large number of polyadenylated nuclear exosome substrates such as snoRNA and miRNA precursors, incompletely spliced mRNAs, and spurious transcripts produced from pseudogenes and intergenic regions. Only a weak accumulation of these exosome substrate targets is observed in *mtr4* mutants, suggesting that MTR4 can contribute, but plays rather a minor role for the degradation of non-ribosomal RNAs and cryptic transcripts in *Arabidopsis*. Consistently, transgene post-transcriptional gene silencing (PTGS) is marginally affected in *mtr4* mutants, but increased in *hen2* mutants, suggesting that it is mostly the nucleoplasmic exosome that degrades aberrant transgene RNAs to limit their entry in the PTGS pathway. Interestingly, HEN2 is conserved throughout green algae, mosses and land plants but absent from metazoans and other eukaryotic lineages. Our data indicate that, in contrast to human and yeast, plants have two functionally specialized RNA helicases that assist the exosome in the degradation of specific nucleolar and nucleoplasmic RNA populations, respectively.

## Author Summary

Cells rely on a number of RNA degradation pathways to ensure correct and timely processing and turnover of both coding and non-coding RNAs. Another important function of RNA degradation is the rapid elimination of misprocessed RNA species, maturation by-products, and nonfunctional RNAs that are frequently produced by pervasive transcription. The main 3′-5′ RNA degradation machine in eukaryotic cells is the exosome, which is activated by cofactors such as RNA helicases. In yeast and human, processing, turnover and surveillance of all nuclear exosome targets depend on a single RNA helicase, MTR4. We show here that the *Arabidopsis* exosome complex can associate with two related RNA helicases, MTR4 and HEN2. MTR4 and HEN2 reside in nucleolar and nucleoplasmic compartments, respectively, and target different subsets of nuclear RNA substrates for degradation by the exosome. The presence of both MTR4 and HEN2 homologues in green algae, mosses and land plants suggest that the functional duality of exosome-associated RNA helicases is evolutionarily conserved in the entire green lineage. The emerging picture is that, despite a high degree of sequence conservation, intracellular distribution, activities and functions of exosome cofactors vary considerably among different eukaryotes.

## Introduction

Efficient processing and degradation of RNA is a key process for the post-transcriptional control of gene expression. The main 3′-5′ RNA degradation machine of eukaryotic cells is the exosome, a multi-subunit complex found in both cytoplasm and nuclear compartments [1], [2]. The exosome participates in a plethora of processing and degradation reactions, including the processing of ribosomal RNAs, snoRNAs and snRNAs, the turnover and quality control of mRNAs and the efficient elimination of RNA maturation by-products and diverse RNA species generated from non-genic regions [3]–[5]. In vivo, exosome activity requires the interaction of the exosome complex with associated RNA helicases. In yeast, cytoplasmic and nuclear exosomes are activated by the RNA helicases SKI2 and MTR4, respectively [6]–[8]. In both yeast and human MTR4 is an essential protein required for all functions of the nuclear exosome [9], [10]. Interestingly, *Arabidopsis thaliana* has two MTR4 homologues, designated AtMTR4 and HEN2 [11]–[13]. We have previously shown that AtMTR4 (encoded by At1g59760) is a predominantly nucleolar protein required for the efficient degradation of misprocessed 5.8S rRNA precursors and specific fragments of the 5′ external transcribed spacer (5′ ETS), a by-product released during processing of three rRNAs from their common precursor transcript [13]. The requirement for AtMTR4 in efficient rRNA production is reflected by the phenotype of *mtr4* mutants, which show a characteristic combination of developmental growth defects also observed in ribosomal protein mutants and in other *Arabidopsis* mutants lacking putative ribosome biogenesis factors such as nucleolin [14]–[19].

*HEN2* (*HUA enhancer 2*, At2g06990) was originally identified in a genetic screen for mutations that enhance the flower morphology defects observed in *hua1* and *hua2* mutants [12]. A follow-up study showed that *hen2* single mutants accumulate, as compared to wild type plants, slightly higher levels of a polyadenylated transcript comprising the two first exons and a large portion of the second intron of the *AGAMOUS* gene product, suggesting that the HEN2 protein could be involved in the degradation of misprocessed *AGAMOUS* transcripts [20]. These data and the strong homology with the exosome activator MTR4 prompted us to examine possible roles of HEN2 in exosome-mediated RNA degradation in *Arabidopsis thaliana*. In contrast to AtMTR4, HEN2 is not required for processing or degradation of 5.8S rRNA precursors or the elimination of the 5′ ETS [13]. We show here that HEN2 is a nucleoplasmic protein that is associated with the *Arabidopsis* exosome core complex and has a specific role in the exosome-mediated degradation of non-coding RNAs, misprocessed mRNAs, introns and transcripts derived from retrotransposons and non-genic regions. Interestingly and as recently reported for human MTR4 [9], [21], HEN2 associates with homologues of the NEXT (for Nuclear Exosome Targeting) complex components and also co-purifies with the cap-binding complex. MTR4, by contrast, is associated with a distinct set of proteins, many of which appear to be involved in ribosome biogenesis. Our results indicate a high degree of spatial and functional specialization of exosome activating RNA helicases in *Arabidopsis*.

## Results

### Both ATMTR4 and HEN2 are associated with plant exosome complexes

**Figure 1:**
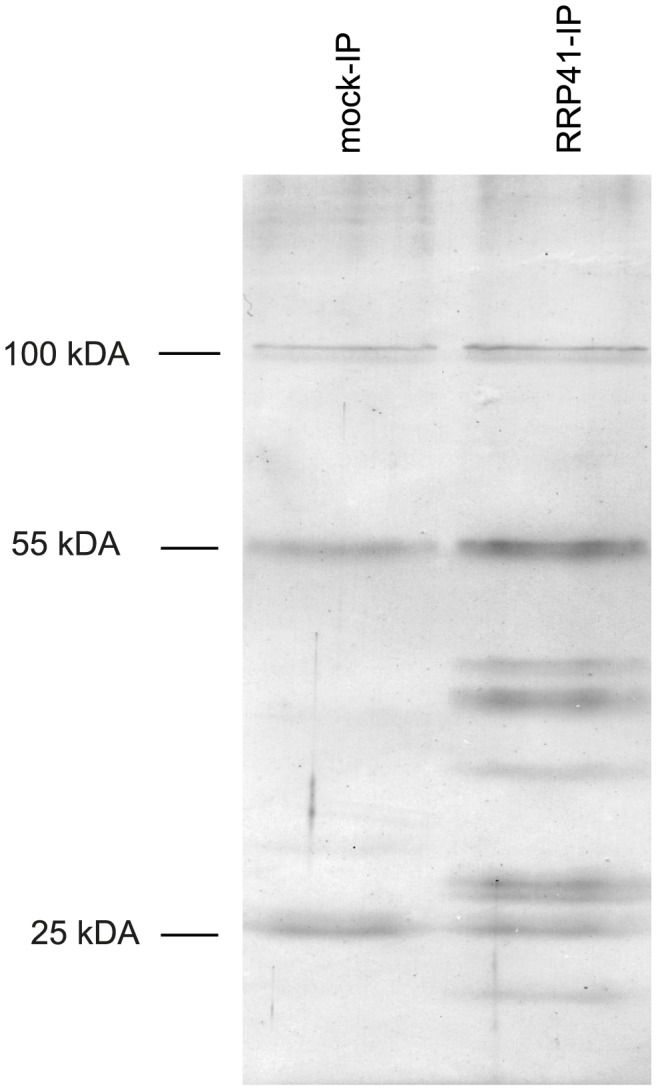
Purification of exosome complexes. Silver-stained SDS-PAGE of proteins co-immunoprecipitated with RRP41-myc. Similar results were obtained when GFP-tagged RRP41 was used as bait. The three main bands observed in mock IP and also present in RRP41-myc IP correspond to immunoglobulins.

Several co-factors of the *Arabidopsis* exosome such as RRP6L1 (RRP6-LIKE 1), RRP6L2, MTR4 and DIS3/RRP44 have been identified based on sequence homology with yeast counterparts and are genetically linked with nuclear exosome functions [13], [22]–[25]. However, none of them has yet been shown to physically interact with the exosome. To better define the composition of plant exosome complexes, we used myc-tagged and GFP-tagged versions of the exosome core subunit RRP41 as baits in co-immunoprecipitation (IP) experiments. RRP41 fusion proteins were expressed under the control of the 1 kb genomic region upstream of the endogenous *RRP41* gene. Both myc-tagged RRP41 and GFP-tagged RRP41 constructs were able to complement the otherwise lethal *rrp41-X* null mutation. A similar full phenotypic complementation was previously reported for TAP-tagged RRP41 [5]. Complementation of the null mutation by both RRP41-myc and RRP41-GFP suggested that both fusion proteins can be integrated in the core exosome complex. To test this hypothesis and identify potential exosome co-factors, tagged-RRP41 and associated proteins were affinity-purified using superparamagnetic particles coated with anti-myc or anti-GFP monoclonal antibodies, respectively. As shown for myc-tagged RRP41 IP, a specific group of proteins were visualized on silver-stained SDS-PAGE gel as compared with mock IP (Fig. 1). The proteins co-purifying with RRP41 were identified by mass spectrometry (nano LC-MS/MS) analysis. A final list of 14 proteins was established by excluding proteins present in mock purifications and by crossing the datasets of three biological repeats (Table 1). All 14 proteins were identified with both Mascot and PEAKS DB algorithms with a false discovery rate <1%. An exhaustive list of the peptides shared by the three RRP41 IPs is shown in Table S1.

**Table 1:** Proteins co-purified with tagged RRP41 and identified by LC-MS/MS.

			Experiment 1		Experiment 2		Experiment 3	
Accession	Name	Function	Mascotscore	#Spectra	Mascot score	#Spectra	Mascot score	#Spectra
AT3G61620	RRP41 (bait)	EXO9	2795.5	232	1458.4	124	1104.2	84
AT3G07750	RRP42	EXO9	2682.2	230	1786.9	134	981.8	59
AT5G38890	CSL4	EXO9	2338.6	221	1446	150	695.8	46
AT1G60080	RRP43	EXO9	2733.2	193	1699.7	118	1105.3	58
AT3G60500	RRP45B/CER7	EXO9 (*)	3290.3	160	1528.4	138	1138.7	53
AT2G25355	RRP40	EXO9	2567.2	146	1584.9	81	723.4	26
AT4G27490	MTR3	EXO9	1992.3	126	1410.7	133	591.8	30
AT1G03360	RRP4	EXO9	1586.5	108	988.5	73	882.3	46
AT3G46210	RRP46	EXO9	1160	86	655.8	68	254.1	14
AT3G27670	RST1	ARM repeat	996.1	36	146.2	4	270.8	7
AT3G12990	RRP45A	EXO9 (*)	836.8	34	450.4	20	283.6	12
AT1G59760	MTR4	RNA helicase	188.2	6	78.2	1	399	6
AT2G06990	HEN2	RNA helicase	222.6	5	235.2	6	134.1	5
AT2G17510	RRP44/DIS3	Ribonuclease	157.4	2	166.9	4	267.3	6

EXO9, Exosome core complex; ARM repeat, Armadillo-repeat protein; EXO9 (*) alternative isoforms of EXO9 subunit RRP45.

As expected, all nine canonical core subunits of EXO9 (RRP41, RRP42, RRP43, RRP45B, RRP46, MTR3, CSL4, RRP4 and RRP40A) were identified which confirms that we indeed purified intact exosome complexes (Table 1, Table S1). In addition to the nine exosome subunits that were previously characterized [5], five novel proteins were detected, albeit with lower number of spectra reflecting a lower abundance as compared to the canonical EXO9 subunits (Table 1, Table S1).

- An isoform of RRP45B, RRP45A, was unambiguously identified by several discriminating peptides (given in bold in Table S1). In *Arabidopsis*, the RRP45 subunit is encoded by two genes: RRP45A and RRP45B/CER7 [5], [26]. Although single *rrp45a* and *rrp45b* mutants are both viable, a double knock out is lethal [26]. The *rrp45b/cer7* mutant is characterized by a defect in cuticular wax accumulation, which is not observed in *rrp45a* mutants, suggesting specialized functions for both isoforms [26]. Only RRP45B/CER7 was previously detected in EXO9 [5], likely because of its higher expression level. Our results show that both isoforms are incorporated in plant exosome core complexes.
- A protein of unknown function RESURRECTION 1 (RST1) also consistently co-purified with EXO9 (Table 1, Table S1). Interestingly, RST1 is crucial for cuticular wax accumulation, the very same biological process affected by lack of RRP45B [26], [27]. The potential interaction between RST1 and EXO9 is being assessed and is not the focus of this study.
- DIS3/RRP44 was also detected in all three biological replicates indicating that this exoribonuclease can associate with the plant EXO9 core exosome as reported for yeast and animal exosomes [28]–[31]. However, as for the other putative co-factors that we detected by LC-MS/MS, DIS3/RRP44 is not visible on silver-stained gels (Fig. 1) and is detected with only few spectra by LC-MS/MS (Table S1), presumably reflecting a labile association to plant EXO9 under the biochemical conditions used for immunoprecipitation experiments. Since none of the three RRP6L present in *Arabidopsis* was detected in our analysis, DIS3/RRP44 represents so far the only exoribonuclease whose physical interaction with plant EXO9 can be inferred from mass spectrometry data.
- In all three replicates, discriminating peptides indicated that two RNA helicases, AtMTR4 and HEN2, co-purified with EXO9 (Table 1, Table S1). AtMTR4 was previously shown to participate in processing or degradation of ribosomal RNA precursors [13]. HEN2, a close relative of AtMTR4 was also suggested to play a role in RNA degradation [12], [20], but its substrates or its role in exosome-mediated pathways have not been studied yet.

### HEN2 is a plant-specific member of the SKI2/MTR4 RNA helicase family

**Figure 2:**
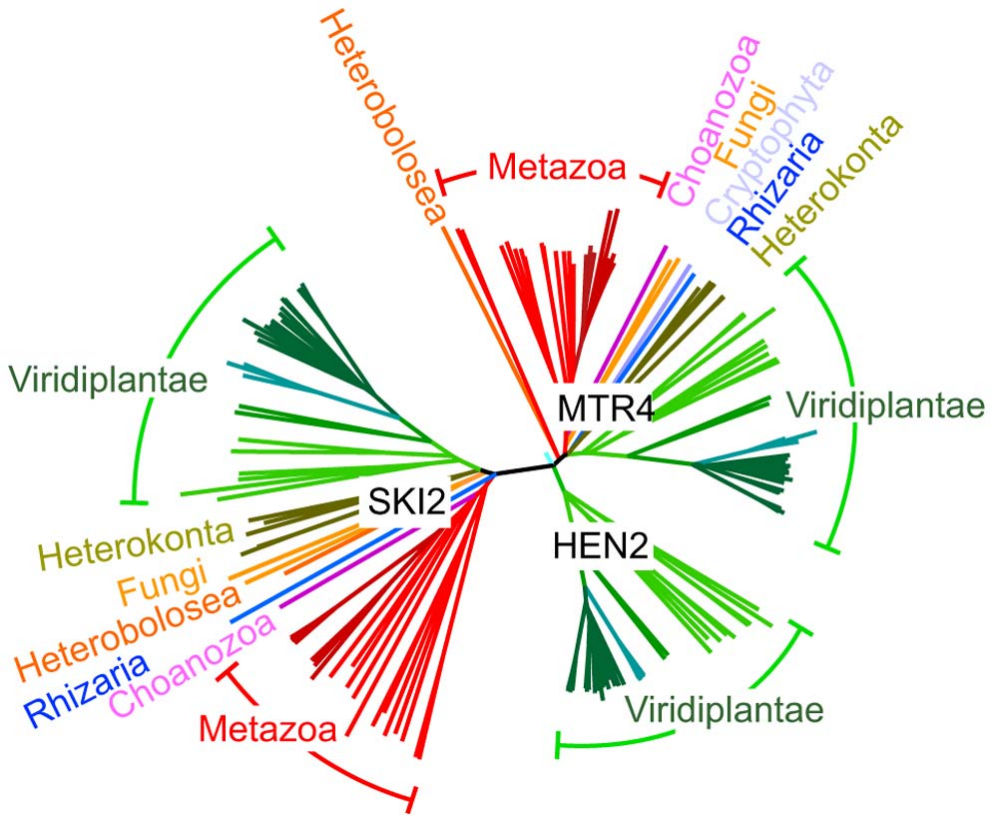
HEN2 proteins are restricted to the green lineage. Phylogenetic tree of the MTR4/SKI family of RNA helicases. The basic branch that separates HEN2 homologues from MTR4 and SKI2 homologues is detected with 1000/1000 bootstraps. Protein sequences were retrieved from metazome, phytozome and JGI databases and aligned with ClustalX. Dark red, vertebrates; light red, other eumetazoa; pink, *M. brevicollis* (Choanozoa); blue, *B. natans* (Rhizaria); orange, *N. gruberi* (Heterobolosea); yellow, *S. cerevisiae* and *S. pombe* (Fungi); olive, *F. cylindrus*, *P. tricornutum*, *T. pseudonana*; *P. cinammomi*, *P. sojae* (Heterokonta); light green, green algae; green, mosses; blue-green, grasses; dark green, dicotyledons. Scale bar = 0.05 amino acid substitutions per site.

AtMTR4 and HEN2 share 43% identity and 59–60% similarity with yeast MTR4 and with each other, but only 24% identity/39% similarity with SKI2, a cofactor of the cytoplasmic exosome [7], [32], [33]. Structural modeling of HEN2 and AtMTR4 confirmed that both possess an arch domain, a characteristic feature of MTR4/SKI2 RNA helicases [34]–[37]. While the modeled structure of HEN2 matches closely to the structure of yeast MTR4, AtMTR4 has an insertion of nine amino acids in the RNA binding part of the arch domain, the KOW-motif [34], [35]. Interestingly, a similar insertion is present in the KOW motifs of all plant MTR4 proteins investigated (Fig. S1). Other characteristic sequence differences between AtMTR4 and HEN2 concern RecA-domains, the arch domain and the C-terminal helix-loop-helix domain (Fig. S2), respectively, and allow the reliable discrimination of HEN2 and AtMTR4 homologues by sequence alignment algorithms. A search for homologues of AtSKI2, AtMTR4 and HEN2 in all genomes available at www.phytozome.net shows that all three RNA helicases are conserved throughout green algae, mosses and land plants. A phylogenetic analysis of related proteins from animals, fungi and other eukaryotic clades revealed that most organisms possess both a single MTR4 and a single SKI2 protein; however, HEN2 homologues are restricted to the green lineage (Fig. 2). Despite of the short insertion in the KOW motif, plant MTR4 proteins cluster with the MTR4 proteins of other organisms, while HEN2 proteins form a separate clade. Taken together, these data suggest that HEN2 is a plant-specific isoform of the nuclear exosome activator MTR4.

### AtMTR4 and HEN2 have distinct intranuclear localization patterns

**Figure 3:**
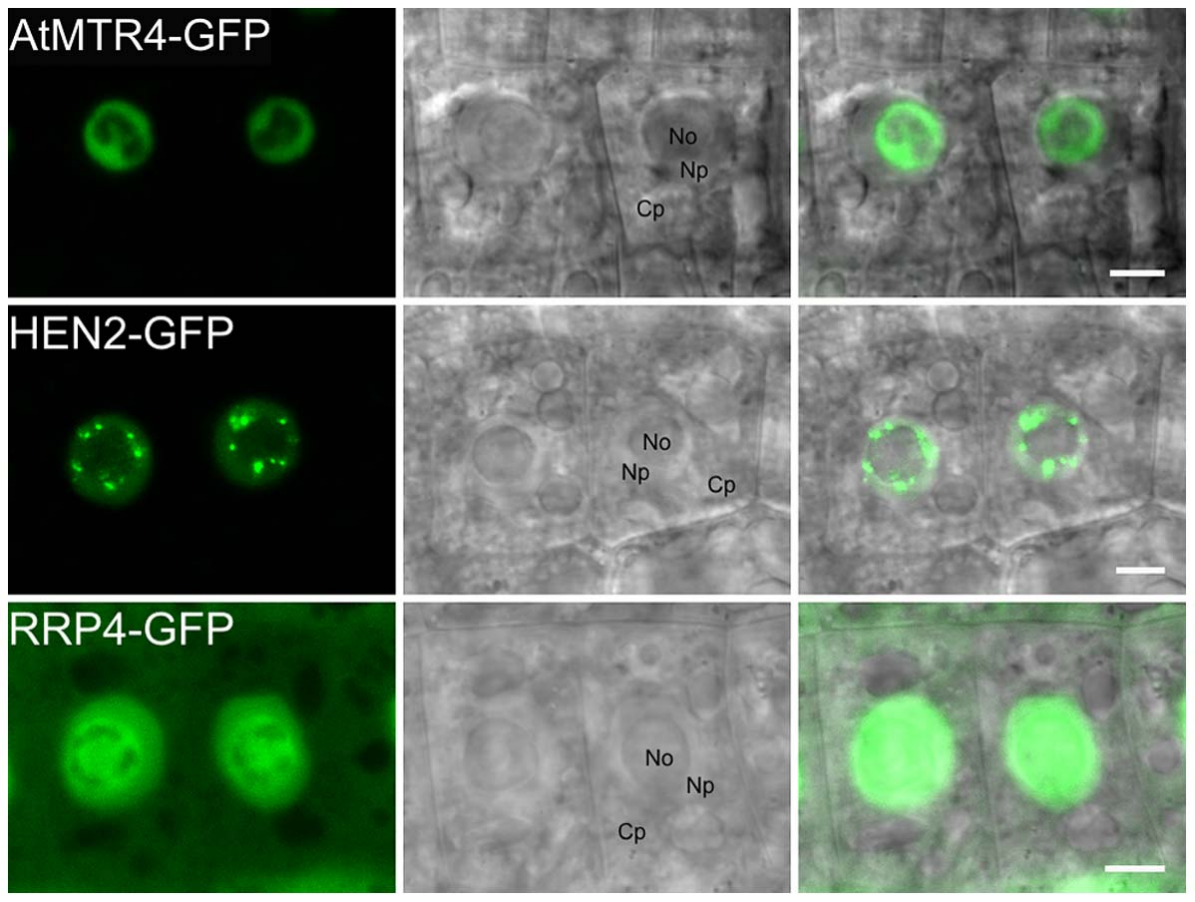
MTR4 and HEN2 have distinct localization patterns. Distribution of GFP-fusion proteins in root cells of stable *Arabidopsis* transformants. No, Nucleolus; Np, Nucleoplasm; Cp, Cytoplasm. Scale bars 5 µm.

To extend previous localization studies [13], we transiently expressed HEN2 and AtMTR4 GFP fusion proteins in *Nicotiana benthamiana* leaves, alongside with RFP-labeled XRN2 and Fibrillarin as nucleolar markers, and with SRP34 as a nucleoplasmic marker [38]–[43]. Similar to XRN2-RFP and Fibrillarin-RFP, AtMTR4-GFP was detected in the nucleus, strongly enriched in nucleoli (Fig. S3). HEN2-GFP and SRP34-RFP were detected only in the nucleoplasm (Fig. S4). Next, we determined the intracellular localization of HEN2-GFP in root tips of stable *Arabidopsis thaliana* transformants. For comparison, we examined roots of plants expressing either AtMTR4-GFP or the exosome core subunits RRP4-GFP and RRP41-GFP. As expected, RRP4-GFP and RRP41-GFP were observed in both cytoplasm and nuclei, and enriched in nucleoli (Fig. 3, Fig. S5). As reported before, AtMTR4-GFP was observed predominantly in nucleoli, and only a faint signal was observed in the nucleoplasm (Fig. 3, Fig. S5) [13]. HEN2-GFP was observed in the nucleoplasm, was enriched in nuclear foci and appeared excluded from nucleoli (Fig. 3, Fig. S5, Fig. S6). These results show that HEN2 is a nucleoplasmic protein, and that AtMTR4 and HEN2 are for the most part located in distinct subnuclear compartments.

### AtMTR4 and HEN2 interact with different sets of proteins

To investigate whether AtMTR4 and HEN2 are associated with specific proteins reflecting their distinct localization and to confirm that both helicases interact with EXO9, we performed IP experiments using plant lines expressing GFP-tagged versions of AtMTR4 or HEN2 in their respective mutant backgrounds. The list of proteins co-purifying with AtMTR4-GFP or HEN2-GFP was established by considering only proteins that were not identified in mock purifications and common to replicate IPs for AtMTR4-GFP and HEN2-GFP experiments, respectively (Tables 2 and 3; Tables S2 and S3).

**Table 2:** Proteins co-purified with MTR4-GFP and identified by LC-MS/MS.

			Experiment 1		Experiment 2		Experiment 3	
Accession	NAME or Annotation	Function	Mascot score	#Spectra	Mascot score	#Spectra	Mascot score	#Spectra
AT1G59760	MTR4 (bait)		7666.5	1332	7928.1	1352	8755.6	1999
AT3G21540	Transducin family/WD-40 repeat protein	Ribo. bio.	537.8	26	606.2	29	720.0	28
AT1G15440	Periodic tryptophan protein 2	Ribo. bio.	718.5	27	863.7	27	857.8	26
AT3G61620	RRP41	EXO9	548.2	21	503.5	21	592.3	26
AT1G63810	Nrap family, nucleolar protein 6 alpha	Ribo. bio.	563.3	18	449.4	10	907.6	26
AT5G11240	Transducin family/WD-40 repeat protein		653.5	24	647.8	24	730.1	24
AT3G06530	U3 small nucleolar RNA-associated protein 10 domain	Ribo. bio.	476.1	17	597.5	19	762.8	24
AT4G04940	Transducin family/WD-40 repeat protein	Ribo. bio.	692.4	26	576.2	22	713.4	23
AT5G38890	CSL4	EXO9	360.8	14	507.5	23	545.0	22
AT3G07750	RRP42	EXO9	580.3	23	487.8	19	576.4	22
AT4G07410	POPCORN	Auxin signaling	464.9	12	430.6	17	618.9	21
AT2G18900	Transducin family/WD-40 repeat protein		468.6	15	371.5	14	639.3	21
AT3G57940	unknown protein, DUF1726	Nuc. bind.	491.5	11	577.3	19	737.6	19
AT5G16750	TORMOZ EMBRYO DEFECTIVE	Ribo. bio.	486.4	8	611.3	13	728.9	19
AT5G22100	RNA cyclase family protein	Ribo. bio.	443.5	12	441.1	16	518.3	17
AT2G25355	RRP40	EXO9	429.7	15	363.8	11	505.8	17
AT4G27490	MTR3	EXO9	355.5	13	331.3	15	342.5	15
AT1G10490	unknown protein, DUF1726	Nuc. bind.	454.7	11	479.9	14	589.9	15
AT1G60080	RRP43	EXO9	565.1	15	360.3	12	558.1	15
AT1G03360	RRP4	EXO9	228.7	7	192.2	5	361.8	15
AT4G05410	YAOZHE	Ribo. bio.	441.3	15	422.7	13	372.9	13
AT3G60500	RRP45B/CER7	EXO9	341.9	12	267.2	7	474.8	12
AT1G63780	IMP4, putative U3 small nucleolar ribonucleoprotein	Ribo. bio.	268.3	9	272.9	11	307.7	11
AT4G28450	WD-40 repeat protein	Nuc. bind.	251.6	11	312.6	10	285.1	11
AT2G47990	SLOW WALKER1	Ribo. bio.	293.0	7	266.0	6	358.5	11
AT1G06720	BMS1 domain protein	Ribo. bio.	269.4	5	319.4	4	430.2	10
AT4G30990	ARM repeat family protein		296.8	7	334.0	6	348.0	9
AT3G10530	Transducin/WD40 repeat protein	Nuc. bind.	265.2	7	249.1	5	298.7	9
AT4G28200	U3 small nucleolar RNA-associated protein 6 family	Ribo. bio.	338.9	7	293.3	6	285.9	6
AT3G46210	RRP46	EXO9	136.5	5	147.9	8	154.2	5
AT5G30495	Fcf2 pre-rRNA processing protein	Ribo. bio.	90.1	3	113.1	6	138.8	5
AT2G36930	Zinc finger (C2H2 type) family protein	Nuc. bind.	75.9	1	70.8	4	108.2	5
AT5G15750	Ribosomal protein S4 family protein	Ribo. bio.	178.6	4	255.0	8	237.8	4
AT1G69070	Nop14-like protein	Ribo. bio.	282.6	6	112.5	3	241.9	4
AT2G46230	PIN domain-like family protein	Ribo. bio.	120.1	5	42.2	1	105.0	4
AT1G18850	unknown protein		141.1	3	96.4	1	181.5	4
AT1G30880	unknown protein		128.2	4	139.5	3	126.4	3
AT4G02400	U3 ribonucleoprotein (Utp) family protein	Ribo. bio.	157.7	4	98.1	2	119.9	3
AT1G31660	ESSENTIAL NUCLEAR PROTEIN 1	Ribo. bio.	163.9	6	66.4	1	139.1	3
AT1G15420	contains small-subunit processome Utp12 domain	Ribo. bio.	80.8	2	64.0	1	112.7	3
AT4G25340	FK506 BINDING PROTEIN 53	Ribo. bio.	112.2	3	60.9	1	64.4	2
AT3G57000	nucleolar essential protein-related, EMG1/NEP1 domain	Ribo. bio.	130.7	3	77.4	4	39.2	1
AT2G17250	NUCLEOLAR COMPLEX ASSOCIATED 4	Ribo. bio.	50.6	3	119.7	4	43.8	1
AT2G03820	NONSENSE-MEDIATED MRNA DECAY 3	Ribo. bio.	35.2	1	67.7	1	110.3	1

Ribo. bio., predicted or known function in ribosome biogenesis; EXO9, subunit of exosome core complex, Nuc.bind., Nucleotide binding.

**Table 3:** Proteins co-purified with HEN2-GFP at 50 mM and 150 mM ionic strength, and identified by LC-MS/MS.

50 mM ionic strength			Experiment 1		Experiment 2	
Accession	Name	Function	Mascot score	#Spectra	Mascot score	#Spectra
AT2G06990	HEN2 (bait)		2663.5	207	3365.9	209
AT5G38600	ZCCHC8 homologue	NEXT	893.2	36	953.4	40
AT3G61620	RRP41	EXO9	427.0	19	530.1	17
AT2G13540	CBP80	CBC	507.3	12	444.9	14
AT4G10110	RBM7 homologue	NEXT	290.5	16	417.7	11
AT3G07750	RRP42	EXO9	338.8	11	528.1	10
AT5G38890	CSL4	EXO9	327.2	10	341.9	9
AT3G60500	RRP45B/CER7	EXO9	319.9	10	369.7	8
AT4G27490	MTR3	EXO9	224.6	8	337.1	5
AT2G25355	RRP40	EXO9	179.3	7	337.6	5
AT1G03360	RRP4	EXO9	138.1	5	199.8	5
AT1G60080	RRP43	EXO9	263.7	4	257.1	5
AT3G12990	RRP45A	EXO9	126.4	4	166.0	3
AT5G44200	CBP20	CBC	79.1	2	49.1	2
AT3G46210	RRP46	EXO9	58.3	3	100.4	1
AT1G67210	ZCCHC8 homologue	NEXT	68.2	1	88.1	1
AT1G02140	MAGO	EJC	62.3	1	100.2	1

150 mM ionic strength			Experiment 3		Experiment 4	
Accession	Name	Function	Mascot score	#Spectra	Mascot score	#Spectra
AT2G06990	HEN2 (bait)		3360.3	315	3290.0	362
AT5G38600	ZCCHC8 homologue	NEXT	842.8	35	877.7	41
AT4G10110	RBM7 homologue	NEXT	428.3	29	380.2	19
AT1G67210	ZCCHC8 homologue	NEXT	119.5	2	139.0	1

EXO9, Exosome core complex; NEXT, putative Nuclear EXosome Targeting Complex; CBC, Cap-Binding Complex; EJC, Exon-Junction Complex.

All canonical nine subunits of EXO9 were identified in both AtMTR4-GFP and HEN2-GFP datasets, which confirmed that both RNA helicases interact with the *Arabidopsis* exosome complex. Remarkably, EXO9 subunits were the sole common proteins among the 43 and 16 significant proteins present in AtMTR4-GFP and HEN2-GFP IPs, respectively (Tables 2 and 3). A Gene Ontology (GO) analysis for the 34 proteins that were specifically co-purified with AtMTR4-GFP exposed that the most significant biological process GO term was ribosome biogenesis (Benjamini-Hochberg corrected p-value, 1.3E-12), which tagged 9 out of 34 proteins. Further data mining revealed that additional 12 proteins have a proven or predicted role in ribosome biogenesis (Table 2). Nine out of the 13 remaining proteins corresponded to transducin/WD40 repeat proteins and/or proteins involved in nucleic acid metabolism (Table 2, Table S2). These results are in agreement with our previous results [13] and further substantiate the role of AtMTR4 in maturation and/or degradation of ribosomal RNA.

HEN2-GFP co-purified the nine canonical subunits of EXO9, the alternative subunit RRP45A and 6 additional proteins (Table 3, Table S3). One of the six proteins that co-purified with HEN2-GFP was a homologue of the exon junction complex (EJC) component MAGO NASHI. Two proteins were the subunits of the cap binding complex (CBC), CBP80 (AT2G13540) and CBP20 (AT5G44200). Finally we identified three putative RNA binding proteins, two of which encoded by *AT5G38600* and *AT4G10110* had high spectral counts (Table 3, Table S3). AT5G38600 is a 532 amino acid protein containing the CX2CX4HX4C zinc-knuckle motif (Pfam14392), particularly found in plant proteins [44]. A BLAST analysis against the human proteome identified ZCCHC8 as the best sequence homologue (E-value 4e-26). ZCCHC8 is a zinc-knuckle protein that was recently identified as part of the human Nuclear EXosome Targeting (NEXT) complex [9]. At4G10100 is a small protein of 173 amino acids that shares some similarity is related to the second component of the human NEXT complex, RBM 7, although the similarity is restricted to the N-terminal two-thirds of the 266 amino acids of RBM 7 (26% identity, 27% similarity for the aligned sequence, Fig. S7). To further check whether HEN2, AT5G38600 and AT4G10110 form a NEXT-like complex in *Arabidopsis*, we slightly increased the stringency of HEN2 immunoprecipitation conditions. By a modest increase of ionic strength from 50 to 150 mM NaCl, the co-purification of EXO9 with HEN2-GFP was lost. However, both AT5G38600 and AT4G10110 were still present in duplicate immunoprecipitations (Table 3, Table S3). Interestingly, a third RNA-binding protein, AT1G67210, was identified in all four HEN2 IPs, albeit with a lower spectral count (Table 3, Table S3). As AT5G38600, AT1G67210 also contains the CX2CX4HX4C zinc-knuckle motif (Pfam14392) and both proteins share 49.6% identities and 57.6% similarities. These results support the existence of a NEXT-like complex in *Arabidopsis* and raise the interesting possibility that multiple NEXT-like complexes exist in plants.

Taken together, our data show that AtMTR4 and HEN2 are associated with distinct sets of proteins. AtMTR4 co-purifies with the exosome, and with putative ribosome biogenesis factors, which highlights the function of AtMTR4 in pre-rRNA processing and degradation. HEN2 co-purifies with the exosome, the CBC complex and with two types of RNA-binding proteins to form a putative plant NEXT-like complex. These data suggest that the functional link between exosome, CBC and NEXT complexes that was recently established in human cells [21] may be conserved in plants. Furthermore, these results strengthen the evidence for a functional specialization of HEN2 and MTR4.

### A selection of known exosome substrates accumulates in *hen2* mutants

**Figure 4:**
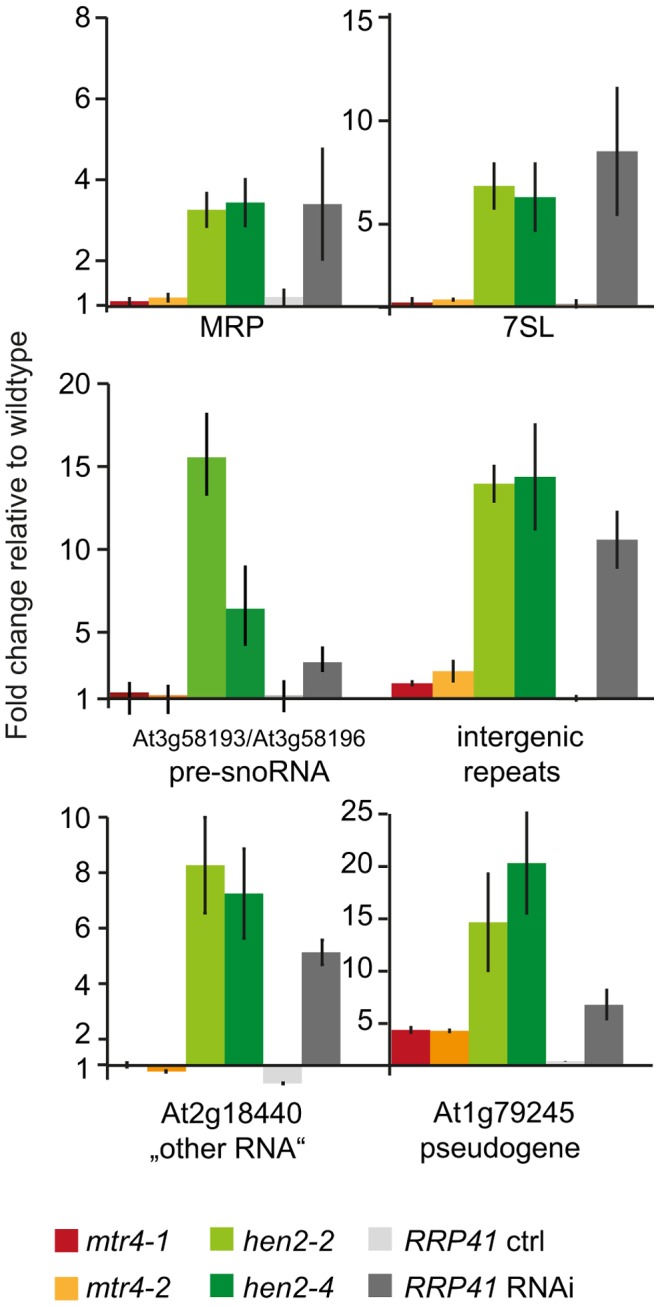
Loss of HEN2 results in over-accumulation of selected exosome targets. Steady-state levels of exosome targets selected from [5], (see also Fig. S8) in *hen2* and *mtr4* mutant seedlings were determined by qRT-PCR. Samples from an inducible *RRP41* RNAi line grown in absence (*RRP41* ctrl) or presence (*RRP41* RNAi) of estradiol were included as controls. The histogram shows the fold change relative to wild type. *mtr4-1* in red, *mtr4-2* in orange, *hen2-2* in light green, *hen2-4* in dark green, *RRP41* control in light grey, *RRP41* RNAi in dark grey. Error bars = SD in three biological replicates.

So far, our data suggested that HEN2 might operate as cofactor of the nucleoplasmic exosome complex. In order to investigate the function of HEN2 for the degradation of exosome substrates, we tested the accumulation of a pseudogene and five non-coding RNAs selected from the list of known polyadenylated plant exosome substrates [5]. Targets comprised the pseudogene At1g79245, the non-coding RNAs MRP and 7SL, the dicistronic precursor of snoRNAs At3g58193 and At3g58196, a non-coding RNA encoded by At2g18440, and intergenic transcripts generated from a repeat region located on chromosome 5 [5]. Additional information and hyperlinks to visualize the selected regions on the SALK transcriptome/exosome website (http://signal.salk.edu/) are provided in Fig. S8. Steady-state levels of the six selected exosome targets were determined by quantitative RT-PCR using oligo-dT primed cDNA samples prepared from seedlings of wild type, *mtr4*-*1*, *mtr4*-*2*, *hen2-2* or *hen2-4* mutant plants. We included also samples from *RRP41* RNAi lines in which depletion of the exosome core subunit RRP41 is triggered by an inducible RNAi construct [5]. As shown in Fig. 4, all exosome substrates tested in this experiment were over-accumulated in *hen2* samples as compared to wild type samples. By contrast, no or only a mild accumulation was observed in *mtr4* mutants. These data provided a first indication that HEN2 is involved in the degradation of nuclear exosome targets that are not substrates of AtMTR4.

### HEN2 participates in mRNA surveillance

To evaluate the respective contribution of HEN2 and AtMTR4 to the degradation of nuclear exosome substrates in an unbiased manner, we determined the accumulation of polyadenylated transcripts using full-genome (tiling) microarray arrays. For this experiment, cDNA was prepared from two biological replicates of wild type, *mtr4-1* and *hen2-4* mutants. Each mutant sample was co-hybridized against a wild type sample to NimbleGen *A. thaliana* 732K whole genome microarrays. The microarray chip contains 1,434,492 strand-specific probes covering both coding and non-coding regions with an average resolution of 175 nt. Probes are 45–85 nt long and designed to achieve a constant Tm of ∼76°C to enhance hybridization consistency across probes. For each biological replicate, expression of each mutant was compared to the expression of the wild type. The statistical analysis, based on a 4-state Hidden Markov Chain, classified probes into four clusters corresponding to over-expressed probes, under-expressed probes, probes with unchanged expression, and noise (not expressed probes), respectively. Interestingly, the analysis did not declare any probe as under-expressed in both biological replicates. This result is in line with the prediction that loss of HEN2 and AtMTR4 impairs RNA degradation, and therefore results predominantly in an increased accumulation of RNA substrates. Indeed, signals for 1860 unique probes were significantly increased in both biological replicates of *hen2* samples. 499 probes were identified as overexpressed in both biological replicates of *mtr4* samples. A file allowing the visualization of the upregulated probes aligned to the *Arabidopsis* genome can be found in dataset S1. For the further analysis, we sorted the probes according to their genome coordinates to identify upregulated regions. Only regions with at least two consecutive probes were considered for interpretation. Upregulated regions were then grouped with respect to annotated features taking into account both TAIR10 annotated genes and recently identified genes encoding snoRNAs, miRNAs and lincRNAs [45]–[47]. This procedure identified 387 regions, the majority of which was upregulated exclusively in *hen2* samples (Tables S4, S5, S6, S7, S8, S9, S10, S11).

**Figure 5:**
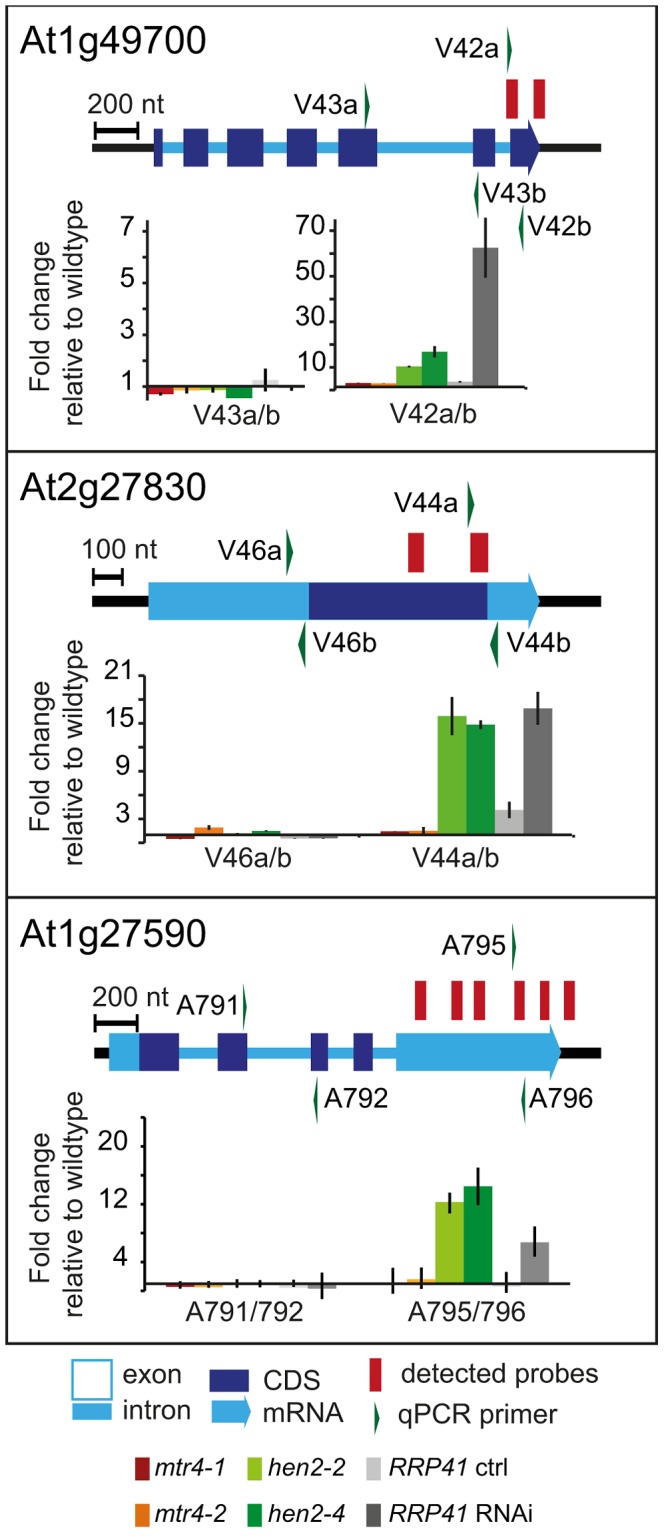
*hen2* mutants accumulate short transcripts derived from mRNA genes. qRT-PCR analysis. A diagram of the genomic locus indicated by the respective AGI number is shown at the top of each panel. Annotated mRNA genes are represented as arrows with dark blue boxes for the CDS, light blue boxes for 3′ and 5′ UTRs, and a light blue line for introns. Red bars above the diagram represent probes detected in the tiling analysis. Green arrows above or below the diagram depict the location of qRT-PCR primers. The corresponding qRT-PCR results for each primer pair are given as fold-change relative to WT in the histograms below each diagram. *mtr4-1* in red, *mtr4-2* in orange, *hen2-2* in light green, *hen2-4* in dark green, *RRP41* control in light grey, *RRP41* RNAi in dark grey. Error bars = SD in three biological replicates.

**Figure 6:**
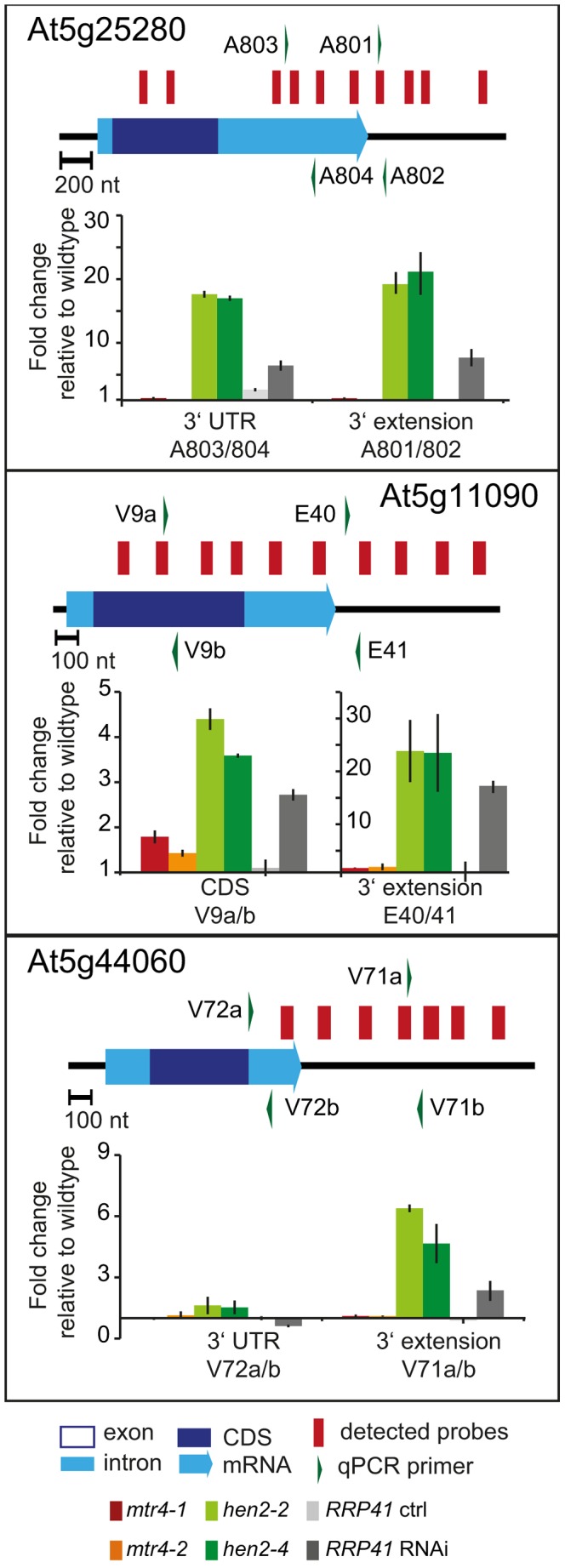
Accumulation of 3′ extended mRNAs in *hen2* mutants. qRT-PCR experiments to test the upregulation of regions that mapped within the body and beyond annotated 3′ ends of mRNAs. Please see legend of Fig. 5 for a detailed explanation of the diagrams.

**Figure 7:**
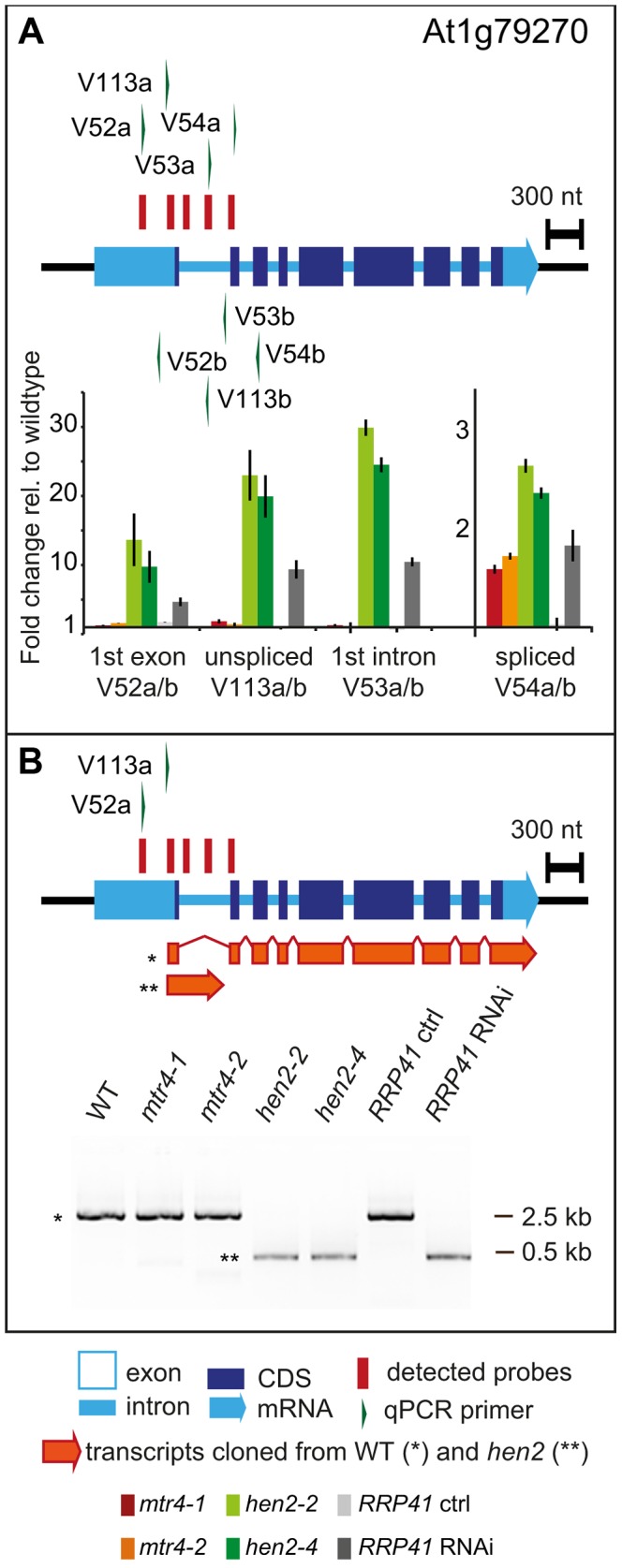
*hen2* mutants accumulate incompletely spliced transcripts. **A** qRT-PCR. The diagram shows the At1g79170 locus. The At1g79170 mRNA is represented as an arrow with dark blue boxes for the CDS, light blue boxes for 3′ and 5‚ UTRs, and a light blue line for introns. Red bars above the diagram represent probes detected in the microarray analysis. Green arrows above or below the diagram depict the location of qRT-PCR primers. The corresponding qRT-PCR results for each primer pair are given as fold-change relative to WT in the histograms below each diagram. Please note the scales. *mtr4-1* in red, *mtr4-2* in orange, *hen2-2* in light green, *hen2-4* in dark green, *RRP41* control in light grey, *RRP41* RNAi in dark grey. Error bars = SD in three biological replicates. **B** 3′ RACE PCR. The diagram shows the At1g79170 locus. Forward primers used for 3′ RACE-PCR on oligo-dT-primed cDNA are shown as green arrows above the diagram. A negative stain of PCR products separated on a 1.5% agarose gel is shown on the bottom. The upper band marked by a star corresponded to the fully spliced mRNA as depicted by the long orange arrow below the diagram. The lower band marked by two stars corresponded to transcripts depicted by the short orange arrow, all of which comprised the unspliced donor site of the first exon/intron junction. The 3′ extremities of these transcripts were located at or upstream of the 3′ acceptor site and were polyadenylated (see Fig. S10).

237 of the upregulated regions mapped to protein coding genes. However, for the majority of the cases, (149 regions, 112 of which were only observed in *hen2* samples, Table S4), the upregulated transcripts were apparently not mature mRNAs. In fact, most of the upregulated regions corresponded to short portions of protein coding genes (Table S4). The upregulation of short regions located in the 3′ portion of protein coding genes was validated by qRT-PCR analysis for three examples (Fig. 5). As a positive control of exosome-mediated RNA degradation, we used the *RRP41* RNAi line. In all three cases, we indeed observed the accumulation of transcripts corresponding to 3′ regions in both the *hen2* and the *rrp41* samples (Fig. 5). Another group of upregulated regions mapped within the body of the transcripts and beyond mature 3′ ends (Fig. 6, Table S4), indicative of alternative 3′ end processing or readthrough transcription. Furthermore, many upregulated regions contained both exonic and intronic sequences, suggesting the accumulation of incompletely spliced transcripts. To test this possibility, we compared by qRT-PCR the steady-state levels of individual exons, introns, regions comprising unspliced intron-exon junctions and correctly spliced transcripts from selected loci (Fig. 7, Fig. S9). These experiments confirmed the overaccumulation of transcripts comprising unspliced donor or acceptor sites in two independent T-DNA insertion alleles of *HEN2* and in *RRP41* RNAi plants (Fig. 7A, Fig. S9). For most loci, we detected the upregulation of both unspliced and spliced transcripts (albeit to different levels, please note the scales in Fig. 7 and S9), suggesting that heterogeneous transcripts are produced and targeted for degradation. In order to map the 3′ extremities of the unspliced transcripts, we amplified and sequenced transcripts derived from the At1g79270 locus by 3′ RACE-PCR (Fig. 7B). cDNA synthesis was initiated by oligo dT, and PCR products were amplified with nested forward primers situated in the 3′ region of the first exon, and the adapter sequence of cDNA synthesis primer as a reverse primer. All PCR products amplified from WT, *mtr4* or *RRP41* control plants corresponded to the fully spliced mature mRNA (Fig. 7B). By contrast, a smaller product of only about 500 bp was amplified from *hen2* or *RRP41* RNAi plants, and corresponded to a population of transcripts that comprised the unspliced donor site of the first intron (Fig. 7B, Fig. S10), while 3′ extremities were close to the acceptor site. Of the 20 clones that were obtained from the *hen2-4* sample 18 were polyadenylated at or close to the intron acceptor site (Fig. S10), indicating that they are indeed marked for degradation by the nuclear exosome. The remaining 2 clones had polyadenylation sites 8 and 52 nt upstream of the acceptor site and likely represent degradation intermediates (Fig. S10). Hence, both qRT-PCR and 3′ RACE results confirmed the results of the microarray analysis and show that polyadenylated transcripts with incorrectly spliced introns accumulate in *hen2* plants.

**Figure 8:**
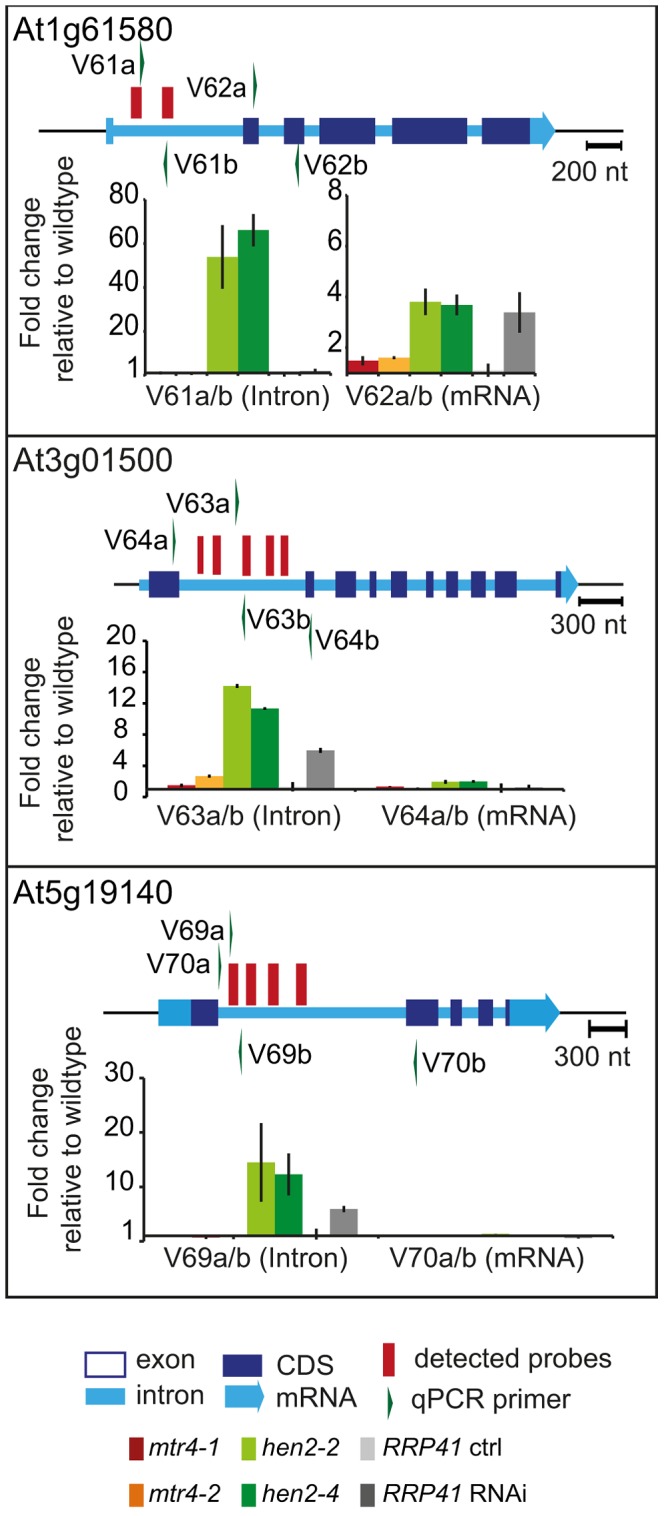
*hen2* mutants accumulate regions of excised introns. qRT-PCR experiments to validate the upregulation of regions located within introns. Primers pairs V62a, V64a/b and V70ab are exon spanning and produce amplicons only from spliced mRNAs under the conditions used for qRT-PCR. Please refer to the legend of Fig. 5 for a detailed explanation of the diagrams.

The tiling data suggested that HEN2 also participates in the elimination of excised introns. In fact, 34 of the 237 protein coding regions that were detected in the microarray analysis corresponded exclusively to intron regions (Table S5), 28 of which were only observed upon loss of HEN2. As for incompletely spliced transcripts, qRT-PCR experiments confirmed the accumulation of intronic regions in two independent alleles of *hen2* and in *RRP41* RNAi samples (Fig. 8). Finally, 54 regions detected by the tiling analysis likely corresponded to mature mRNAs since they were regions with all probes in exons, and the detected regions spanned at least 50% of the mRNA. 33 mRNAs were detected in *hen2*, 9 mRNAs in *mtr4*, and 12 in both *hen2* and *mtr4* samples (Table S6). The upregulation of the pseudogene At1g79245 (Fig. 4) and several other mRNAs in *hen2* and *mtr4* samples was validated by qRT-PCR (Fig. S11). However, not all of the tested mRNAs were also found upregulated in *RRP41* RNAi samples (Fig. S11). Moreover, none of the positively tested mRNAs was previously identified as an exosome-regulated mRNA [5]. Finally, some mRNAs were only detected in the very same samples that have been used for the microarray, but not in other mutant plants grown in the same culture conditions (Fig. S12). This inconsistence was in sharp contrast to all other types of substrates that were tested in the course of the study, which were reproducibly detected in all replicates, in independent *hen2* T-DNA insertion mutants, and in *RRP41* RNAi lines. Therefore, we doubt that all of the mRNAs that were detected in our tiling analysis represent true substrates of exosome-mediated decay. Although nuclear degradation can probably contribute to mRNA degradation [3], [5], [48], the upregulation of mRNAs can also be explained by indirect effects, e.g. a differential response of WT and mutant plants to growth conditions. Indeed, data mining revealed that many of the mRNAs detected by the tiling arrays are linked to stress response (Table S12). Hence, the majority of the mRNAs that we detected by the tiling array are probably not *bona fide* substrates of HEN2 or the nuclear exosome. By contrast, the upregulation of short mRNA-derived transcripts (Fig. 5, Table S4), 3′ extended mRNAs (Fig. 6, Table S4), unspliced transcripts, (Fig. 7, Table S4, Fig. S9, Fig. S10), introns (Fig. 8, Table S5) is consistently detected in all replicates of both mutant alleles of *hen2* and in *RRP41* RNAi samples, and can be considered as *bona fide* substrates of the nuclear exosome and the RNA helicase HEN2.

Taken together, our data indicate that loss of HEN2 results in the accumulation of short transcripts derived from mRNA regions, 3′ extended transcripts, incompletely spliced mRNA transcripts, and excised introns. The polyadenylated status of the accumulated transcripts indicates that they are tagged for degradation by the exosome, and indeed, all these classes of mRNA-derived transcripts have been described as exosome targets [5]. Hence, the most straightforward explanation for the accumulation of these transcripts in *hen2* mutants is that HEN2 is required for the exosome-mediated elimination of different types of probably unfunctional RNAs that are generated from protein coding genes. Only a small number of such transcripts were observed in *mtr4* mutants (Table S4, S5) and accumulated at lower levels (Fig. 5–8, Fig. S9), indicating that MTR4, as compared with HEN2, has a rather minor contribution to nuclear mRNA surveillance.

### HEN2 is the major RNA helicase for the degradation of non-coding nuclear exosome substrates

**Figure 9:**
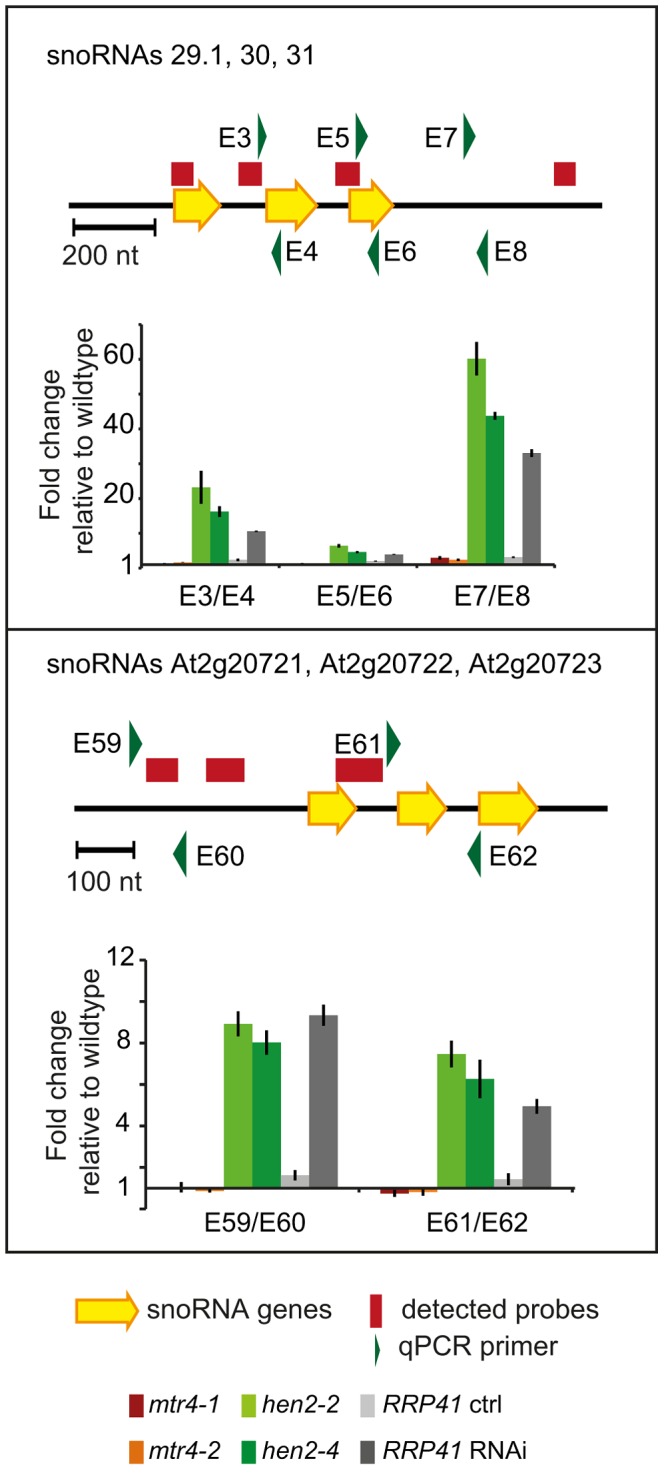
*hen2* mutants accumulate snoRNA precursors. qRT-PCR. A diagram of the genomic locus with the indicated snoRNA genes is shown at the top of each panel. Individual snoRNA genes are represented as yellow arrows. Red bars above the diagram represent probes detected in the tiling analysis. Green arrows above or below the diagram depict the location of qRT-PCR primers. The corresponding qRT-PCR results for each primer pair are given as fold-change relative to WT in the histograms below each diagram. *mtr4-1* in red, *mtr4-2* in orange, *hen2-2* in light green, *hen2-4* in dark green, *RRP41* control in light grey, *RRP41* RNAi in dark grey. Error bars = SD in three biological replicates.

Next, we examined the contribution of HEN2 and MTR4 to the degradation of non-coding transcripts. Of the 387 upregulated regions detected in the microarray analysis, 150 regions mapped to non-coding regions. 9 regions mapped to transposable elements (6 in *hen2*, 3 in *mtr4*) and were not further investigated (Table S7). 45 regions, all of which were exclusively observed in *hen2* mutants, contained one or more snoRNA genes (Table S8), including the regions encoding snoRNAs At3g58193 and At3g58196 (Fig. 4). In fact, almost all of the snoRNA regions detected in our tiling array have also been previously identified as exosome substrates (see Table S8 last column, and [5]). To further investigate the contribution of HEN2 to the degradation of snoRNA precursors, we tested two additional snoRNA regions by qRT-PCR (Fig. 9). The results indicated a strong accumulation of snoRNA precursors in both *hen2* mutant lines and in *RRP41* RNAi samples. The preferential accumulation of snoRNA precursor transcripts was further confirmed by transferring 3′ RACE-PCR products to membranes followed by hybridisation with radiolabelled probes (Fig. S13). These data strongly indicate that HEN2, but not MTR4, plays an important role for the degradation of snoRNA precursors.

**Figure 10:**
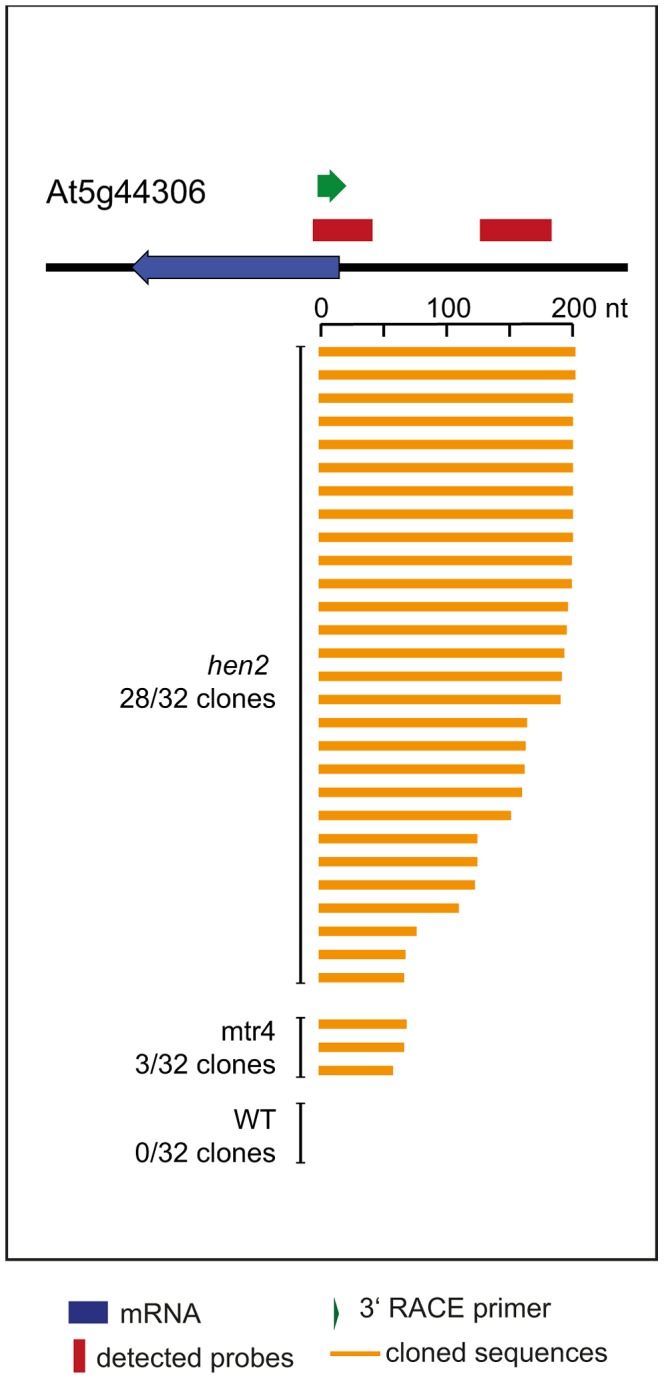
Loss of HEN2 results in accumulation of polyadenylated antisense transcripts. 3′ RACE-PCR. A diagram of the At5g44306 locus is shown at the top. A blue arrow represents the At5g44306 mRNA. Red bars above the diagram represent probes detected in the tiling analysis. The location of the primer used for 3′ RACE PCR is indicated by a green arrow above the diagram. Each of the orange horizontal bars below the diagram represents a polyadenylated clone obtained from the indicated sample. 28 of 32 clones obtained from *hen2* samples and 3 of 32 clones obtained from *mtr4* samples corresponded to antisense transcripts of 67 to 208 nt lenght. 0 of 32 clones obtained from WT samples corresponded to the target sequence.

Other non-coding RNA regions were also unequally distributed between *hen2* and *mtr4* samples. 22 regions in *hen2* samples encoded lincRNAs, putative miRNA precursors or other non-coding RNAs (Table S9), among them a portion of the region encoding GUT15 (for gene with unstable transcript 15)/At2g18440 (Fig. 4). Only 2 of such non-coding RNA regions were observed in *mtr4* samples (Table S9), indicating that MTR4 plays a minor role for the degradation of non-ribosomal non-coding RNAs. Similarly, we detected 29 putative antisense transcripts in *hen2* samples, while only 2 potential antisense regions were upregulated in *mtr4* samples (Table S10). To confirm the polyadenylated status of antisense transcripts, we selected one of the potential antisense regions for 3′ RACE experiments. Cloning and sequencing of the PCR products revealed that 0 of 32 clones obtained from WT samples corresponded to the target sequence (Fig. 10, Fig. S14). By contrast, 28 of 32 clones obtained from *hen2* samples and 3 of 32 clones obtained from *mtr4* samples corresponded indeed to antisense transcripts derived from the target region (Fig. 10, Fig. S14). Antisense sequences were polyadenylated, a hallmark of exosome-mediated RNA degradation, and between 67 and 208 nt long (Fig. 10, Fig. S14). These data strongly suggest that the antisense transcripts derived from the At5g44306 locus are indeed substrates of polyadenylation-mediated decay facilitated by HEN2 and the RNA exosome. Finally, the microarray analysis detected 43 regions without any annotated genome features, including the intergenic repeat region on chromosome five that was already detected by our initial qRT-PCR experiments (Fig. 4). Similar to the distribution of non-coding RNA regions and potential antisense transcripts, the majority of the non-annotated regions (38 of 42) were exclusively observed in *hen2* samples, while only 4 of 42 regions were found in *mtr4* samples. These results indicate that the elimination of spurious transcripts generated from antisense or non-annotated regions that are usually described as the “dark matter” of the transcriptome relies mostly on HEN2.

**Figure 11:**
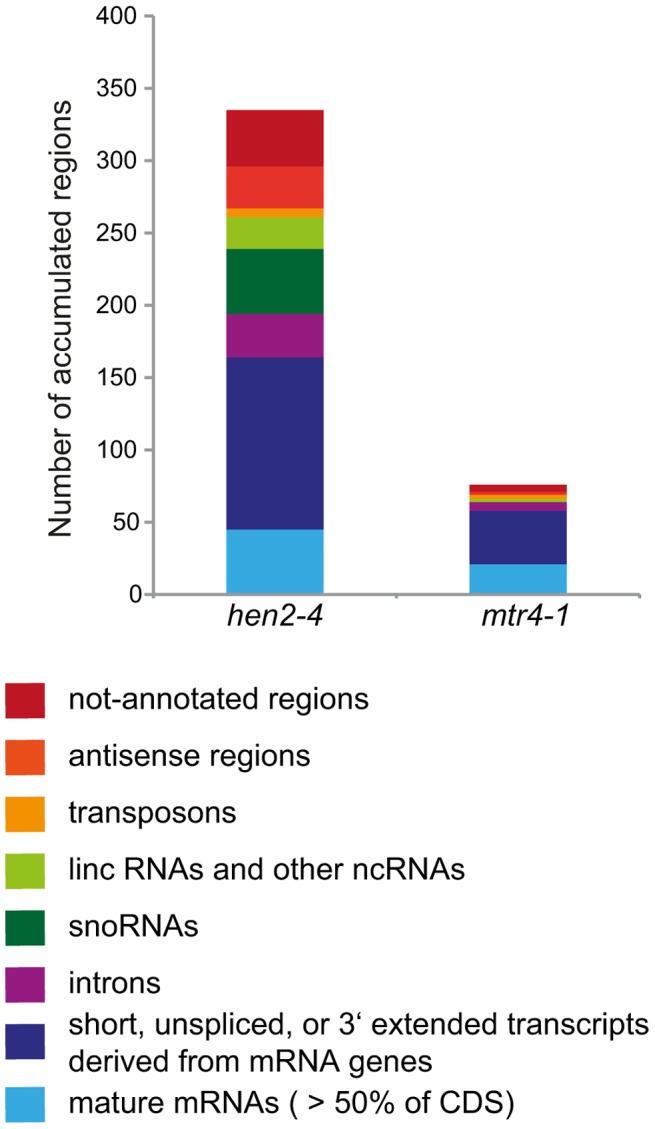
Degradation of non-ribosomal exosome targets depends largely on HEN2. The transcriptomes of WT, *hen2-4* and *mtr4-1* plants were compared by whole genome microarrays. The histogram shows the total number of regions that were, as compared to wild type, overaccumulated in *hen2* or *mtr4* samples, respectively. A complete list of the upregulated regions can be found in Tables S4, S5, S6, S7, S8, S9, S10, S11.

Taken together, the microarray analysis revealed that a large number of exosome targets, including short or incompletely spliced transcripts derived from mRNA genes, precursors and processing by-products of non-coding RNAs, and spurious transcripts generated from antisense and intergenic regions accumulate specifically in *hen2* mutants (Fig. 11). This indicates that HEN2 has a major function in the elimination of many different types of nuclear exosome substrates. A much smaller number of such non-ribosomal exosome substrates accumulated in *mtr4* plants (Fig. 11), and average accumulation levels in *mtr4* samples were lower than in *hen2* samples (Fig. S15). These data indicate that AtMTR4, though it can apparently contribute at least to the degradation of mRNA-derived transcripts, plays a rather minor role in nuclear RNA surveillance.

### HEN2 has an antagonistic effect on post-transcriptional gene silencing mediated by a sense transgene (S-PTGS)

**Figure 12:**
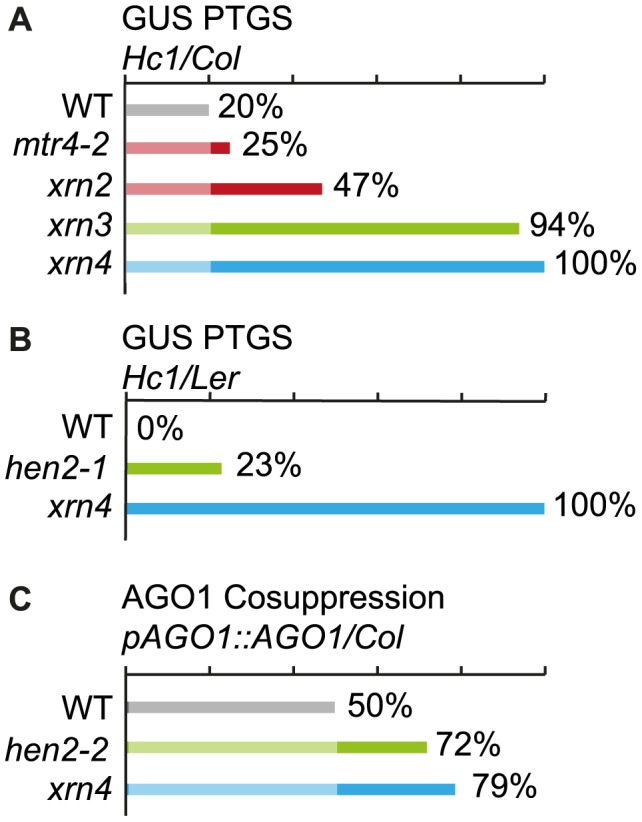
Effect of *mtr4* and *hen2* mutations on posttranscriptional silencing. Diagrams show the proportion of plants that undergo systemic silencing of a GUS (panels A and B) or AGO1 (panel C) reporter transgene in the indicated mutants and backgrounds. The color code indicates the intracellular localization of the mutated proteins: red marks the nucleolar proteins; green marks nucleoplasmic proteins, and blue mark cytoplasmic proteins. A. Loss of MTR4 has only a marginal effect on GUS S-PTGS. The effect of the *mtr4-2* mutation on silencing of a GUS reporter transgene was tested in the Hc1/Col reporter system as described in [51]. For comparison, we included previously published data from [49] that show the effects of compromised 5′-3′ exoribonucleases XRN2 (nucleolar), XRN3 (nucleoplasmic) or XRN4 (cytoplasmic) in the same reporter system. B. Loss of HEN2 triggers silencing of a GUS PTGS reporter. The effect of *hen2-1* (in Ler) on GUS-PTGS was tested in an Hc1-reporter line backcrossed to Ler. Please note that the Hc1/Ler reporter line does not trigger silencing of the GUS reporter in WT. *xrn4* in Hc1/Ler was used as a positive control. C. Loss of HEN2 triggers co-suppression of AGO1. To further confirm the role of HEN2 as silencing suppressor, WT, *hen2-2* and *xrn4* plants (in Col) were transformed with a *pAGO1::AGO1* construct that triggers cosuppression of the endogenous AGO1 gene.

Previous data indicated that mutations in 5′-3′ exoribonucleases in nucleoli (XRN2), nucleoplasm (XRN3) or cytoplasm (XRN4) enhance the efficiency of post-transcriptional gene silencing mediated by sense transgenes (S-PTGS) [49]. Mutations in the exosome core components RRP4 and RRP41, or mutations in the exosome co-factors RRP44/DIS3 and RRP6L1 also enhance S-PTGS, suggesting that both 5′-to-3′ and 3′-to-5′ RNA degradation pathways limit the entry of aberrant transgene RNAs into the S-PTGS pathway [50]. To determine in which nuclear compartment the exosome counteracts transgene PTGS, we analyzed the effect of mtr4 and hen2 mutations on S-PTGS using the Arabidopsis reporter line Hc1 [49]–[52]. Line Hc1 carries a 35S::GUS transgene that triggers S-PTGS at a frequency of 20% at each generation, making this line ideal for identifying mutations that either increase or decrease S-PTGS efficiency. However, only EMS mutants or 35S CaMV promoter-free T-DNA insertion mutants are amenable for such analyses because copies of the 35S CaMV promoter present in the SALK, WISC or GABI T-DNA collections often interfere with the expression of 35S CaMV promoter-driven transgenes, which could report an impact on S-PTGS that is not directly related to the function of the mutated gene [53]. Only the T-DNA *mtr4-2* [13] and the EMS *hen2-1* mutant [12] fit this requirement. *Hc1/mtr4-2* plants triggered S-PTGS at a frequency of 25% (n = 96), which is only slightly higher than S-PTGS frequency in *Hc1* controls (Fig. 12A). These data indicate that compromising exosome activity in nucleoli has only a limited effect on transgene S-PTGS. For comparison, loss of the nucleolar exoribonuclease XRN2 was previously shown to trigger S-PTGS at a frequency of 47% [49] (Fig. 12A). In contrast, compromising the nucleoplasmic 5′-to-3′ exonuclease XRN3 had a stronger effect (Fig. 12A) [49]. To determine if loss of the nucleoplasmic protein HEN2 also affects S-PTGS, the *hen2-1* mutation, which is in the Landsberg erecta (Ler) ecotype, was crossed to an Hc1/Ler line resulting from ten backcrosses of Hc1 to Ler. Remarkably, no *Hc1/Ler* plants exhibited S-PTGS (n = 96, Fig. 12B), suggesting that either L*er* is less prone to trigger S-PTGS than Col or that *Hc1* has lost its capacity to trigger S-PTGS after ten backcrosses to L*er*. This later hypothesis was ruled out by crossing *Hc1/Ler* to *xrn4-1* (in L*er*). Mutations in the cytoplasmic 5′-to-3′ exonuclease XRN4 are known to enhance S-PTGS in Col [49] (Fig. 12A), so S-PTGS was expected to occur in *Hc1/xrn4-1/Ler* plants if the *Hc1* locus has retained its ability to trigger S-PTGS in L*er*. S-PTGS was observed in 100% of *Hc1/xrn4-1/Ler* plants (n = 96, Fig. 12B), indicating that L*er* is less prone to trigger S-PTGS than Col, but that the *Hc1/Ler* line still is amenable to identify mutations that enhance S-PTGS. Indeed, 23% of *Hc1/hen2-1/Ler* plants (n = 83) exhibited S-PTGS (Fig. 12B), indicating that compromising exosome activity in the nucleoplasm strongly enhances transgene S-PTGS. The antagonistic effect of HEN2 on S-PTGS was confirmed in Col using an *AGO1* transgenic reporter system. In this system, transformation of wild type Col with a T-DNA carrying an ectopic *pAGO1::AGO1* construct triggers cosuppression (S-PTGS) of endogenous AGO1 in 50% of the transformants [54] (Fig. 12C). Transformation of *hen2-2* (in Col) with the same *pAGO1::AGO1* construct triggered AGO1 cosuppression in 72% of the transformants (Fig. 12C). This increase is almost comparable to the effect of *xrn4* on AGO1 cosuppression (Fig. 12C), indicating that *hen2* strongly affects S-PTGS in Col. Taken together, these data show that HEN2 counteracts S-PTGS in both Col and L*er*, likely through its role as a co-factor of the nucleoplasmic exosome.

## Discussion

We show here that two isoforms of MTR4, AtMTR4 and HEN2, assist the *Arabidopsis* exosome for the degradation of mostly distinct sets of nuclear RNA substrates. Both AtMTR4 and HEN2 co-purify with the *Arabidopsis* exosome core complex but AtMTR4 and HEN2 occupy primarily distinct intranuclear compartments. The main role of the nucleolar isoform AtMTR4 is to assist in the exosome-mediated degradation of misprocessed rRNA precursors and maturation by-products [13]. Our new data show that the main function of the nucleoplasmic isoform HEN2 is to assist the exosome-mediated processing and/or degradation of snoRNAs and snoRNA precursors, miRNA precursors, lincRNAs, and a large number of spurious transcripts derived from antisense and non-annotated regions. In addition, HEN2 is involved in the degradation of excised introns and incompletely spliced or otherwise mis-transcribed or mis-processed mRNAs. Of the 387 regions detected in this study, 100 have been previously identified as targets of the *Arabidopsis* core exosome (Tables S4, S5, S6, S7, S8, S9, S10, S11) using a different type of tiling microarray [5]. However, our qRT-PCR data indicate that majority of the HEN2 substrates accumulate also in *RRP41* RNAi samples, even though some of them were not detected previously [5], such as At1g79270 (Fig. 6), At3g26510 (Fig. S9) and At1g58602 (Fig. S9). Vice versa, several known exosome substrates were not identified by our tiling analysis but easily detected by qRT-PCR (Fig. S16). These findings indicate that both tiling studies probably underestimate the contribution of HEN2 and the exosome to RNA surveillance. Only a small number of exosome substrates are observed in *mtr4* mutants, which indicates that AtMTR4 participates, but plays only a minor role in nuclear RNA surveillance. This is in line with the finding that AtMTR4 and HEN2 have marginal and strong contributions, respectively, to the exosome activity that likely degrades aberrant transgene RNAs in the nucleus to limit their entry in the PTGS pathway [49], [50].

In a previous study, we have shown that *hen2* mutants do not accumulate the 5.8S rRNA precursors and the 5′ ETS that are observed upon down-regulation of AtMTR4 [13]. Accordingly, *hen2* single mutants do not display any of the developmental defects linked to disturbed ribosome biogenesis or ribosome function that were observed in *mtr4* mutants. These data were the first clues that AtMTR4 and HEN2 have rather distinct functions in plants. However, we were not able to obtain double *mtr4 hen2* mutants, signifying that simultaneous loss of both AtMTR4 and HEN2 is lethal [13]. The data presented here suggest that AtMTR4 can perform some of the functions of HEN2. For instance, a limited number of mRNA-derived fragments, non-coding RNAs and spurious transcripts accumulated in *mtr4* single mutants, indicating that AtMTR4 can contribute, even in presence of HEN2, to the degradation of non-ribosomal exosome targets. In line with this, a small fraction of AtMTR4-GFP can be detected in the nucleoplasm of stable *Arabidopsis* transformants (Fig. S17, see also [13]). Taking together, these results indicate that a limited overlap between AtMTR4 and HEN2 functions exists. However, our data show that most exosome functions are activated either by AtMTR4 or by HEN2 in the nucleolus and the nucleoplasm, respectively.

It is interesting to note that *Arabidopsis* has also specific nucleoplasmic and nucleolar isoforms (RRP6L1 and RRP6L2, respectively) of RRP6, a catalytically active exoribonuclease associated with nuclear exosomes in yeast and human [28], [30]. So far, we and others [5] did not detect any of the plant RRP6-like proteins in plant exosome preparations. However, we have previously shown that the downregulation of the nucleolar isoform RRP6L2 leads to a mild accumulation of misprocessed 5.8S rRNA precursors and the 5′ ETS, suggesting that RRP6L2 acts in the same degradation processes as AtMTR4 [13], [55]. By contrast, we did not detect a significant overaccumulation of HEN2 targets upon down-regulation of the nucleoplasmic isoform RRP6L1 (data not shown). A possible explanation is that RRP6L2 and RRP6L1 can substitute for each other in the degradation of HEN2 targets, since the two nuclear RRP6-like proteins appear to have both specific and common roles linked to the degradation of exosome targets [24], [55]. Interestingly, a recent study revealed that RRP6L1, but not RRP6L2 has also a role in transcriptional silencing by retaining PolV transcripts on chromatin, thereby promoting the production of 24 nt siRNAs that direct DNA methylation via the RdDM pathway [56]. Remarkably, this function of RRP6L1 is independent of the core exosome [56]. By contrast, transcriptional silencing at soloLTR loci is mediated by both RRP6L and the core exosome [24]. In addition, RRP6L1 and the exosome core complex have a common role in 21-nt siRNA-dependent posttranscriptional silencing (PTGS), since downregulation of either RRP41 or RRP6L1 alone is sufficient to enhance PTGS in the sensitive Hc1-GUS reporter system that was used in this study [50]. Hence, the role of RRP6L1 in PTGS is likely linked to exosome-mediated RNA degradation, suggesting that HEN2 and RRP6L1 are involved in at least one similar function.

In animals and fungi, a single MTR4 protein is present in nucleoplasm and nucleoli, and essential for both processing/degradation of rRNA precursors and the elimination of all other nuclear exosome substrates [8]–[10], [57]. However, both yeast and human MTR4 proteins are incorporated in more than one exosome activator/adapter complex. Yeast MTR4 is detected in TRAMP4 and TRAMP5 (for TRF4/5 AIR1/2 MTR4 Polyadenylation), each of which comprises a RNA binding protein and a non-canonical poly(A) polymerase [58]–[61]. Although TRAMP4 and 5 have a similar composition and many redundant functions, TRAMP5 seems be more important for the polyadenylation of pre-rRNAs while TRAMP4 might be more important for the degradation of other non-coding RNAs and intergenic transcripts [62]–[64]. The functional specialization between nucleolar and nucleoplasmic exosome activator complexes is clearer in human. In nucleoli, hMTR4 is incorporated in a TRAMP-like complex which polyadenylates rRNA maturation by-products [9]. In the nucleoplasm, hMTR4 is associated with the NEXT (for Nuclear EXosome Targeting) complex, which targets PROMPTS (PROMoter uPstream TranScripts) for degradation by the exosome [9]. Hence, both yeast and animals possess nucleolar and nucleoplasmic exosome activators, which share MTR4 as a central component. By contrast, an exosome activating system with two specialized RNA helicases has evolved early in the green lineage. Interestingly, both the nucleoplasmic fraction of human MTR4 and the *Arabidopsis* nucleoplasmic-specific RNA helicase HEN2 appear associated with similar RNA binding proteins to form NEXT and NEXT-like complexes, respectively, and with the cap-binding complex [9], [21, this study]. These findings suggest a high degree of functional conservation between the nucleoplasmic fraction of human MTR4 and plant HEN2. By contrast, a TRAMP-like complex comprising a non-canonical poly(A) polymerase remains to be identified in plants. Hence, the emerging picture is that only the core exosome machinery is conserved in all eukaryotes, while exosome-associated activities and activating complexes show intriguing diversity and complexity in fungi, insects, animals and plants.

## Methods

### Plant material

With the exception of *hen2-1* [12] used for the S-PTGS assay, all *Arabidopsis thaliana* plants were of Columbia ecotype (Col-0). T-DNA insertion lines were retrieved from NASC (http://arabidopsis.info/). *mtr4-1*, *mtr4-2*, *hen2-2* and *hen2-4* lines are described in [13]. *RRP41* RNAi lines are described in [5]. The S-PTGS reporter line *Hc1* was first described in [51]. *Hc1/xrn/col* lines are described in [49]. Unless stated otherwise, plants were grown on soil at 20°C with cycles of 16 h light/8 h darkness.

### Sequence analysis and phylogeny

Sequences were retrieved from Phytozome (http://www.phytozome.net), Metazome (http://www.metazome.net) and JGI (http://genome.jgi.doe.gov) genome databases, using AtMTR4, HEN2 and AtSKI2 proteins as BLAST queries. Structures of AtMTR4 and HEN2 were modeled with MODELLER Software (http://modbase.compbio.ucsf.edu/ModWeb20-html/modweb.html) using the crystal structures of *S. cerevisiae* MTR4 (PDB 3L9O and 2XGJ) [34], [35] as templates. Alignments were performed with Chimera (http://www.cgl.ucsf.edu/chimera, for structure-based sequence alignments) and ClustalX (http://www.clustal.org, for phylogenetic analysis). The phylogenetic tree was calculated with the neighbor-joining algorithm built in ClustalX and 1000 bootstraps, and drawn with Figtree (http://tree.bio.ed.ac.uk/software/figtree).

### Expression of GFP and myc-tagged fusion proteins

For the expression of RRP4, AtMTR4 and HEN2 GFP fusion proteins under the control of the 35S CaMV promoter, the coding sequences of RRP4, AtMTR4 and HEN2 were amplified from cDNA and cloned into vector pK7FWG2 [65]. For expression of GFP-tagged or myc-tagged RRP41 the genomic sequence of *RRP41* including 1 kb upstream of the *RRP41* gene was cloned into vectors pGWB604 and pGWB616, respectively [66]. For immunoprecipitations (see below), AtMTR4-GFP was expressed under the control of its own promoter. To do so, a genomic region comprising 1 kb upstream of the MTR4 gene, the first two exons and the first intron were fused to the CDS downstream of the second exon and cloned into pGWB604 [66]. Constructs that allow the expression of RFP-tagged FIB1 [67], XRN2 and SRP43a [68] were kind gifts of Jane Brown and Martin Crespi, respectively. Infiltration of *N. benthamiana* leaves was performed as described in [50] except that P19 was used as a suppressor of silencing. *Arabidopsis* plants were transformed by the floral dip method [69]. Root tips of stable transformants were examined 8 days after germination by confocal microscopy.

### Co-immunoprecipitation and mass-spectrometry analysis

RRP41-myc, RRP41-GFP, AtMTR4-GFP and HEN2-GFP-fusion proteins were extracted from flowers of stable *Arabidopsis* transformants and purified using magnetic microparticles coated with monoclonal myc or GFP antibodies (MACS purification system, Miltenyi Biotech) according to the manufacturer's instructions except that SDS was omitted from washing buffers. Co-IP experiments were carried out in triplicates for RRP41 and MTR4 and duplicates for HEN2 with 50 mM and 150 mM NaCl.

For in-gel digestion, samples were separated by SDS-PAGE followed by trypsic digestion and peptide extraction as described in [70]. Otherwise, proteins were eluted directly from magnetic beads in 1× Laemmli buffer, precipitated with 100 mM ammonium acetate in methanol, and resuspended in 50 mM ammonium bicarbonate. After reduction and alkylation steps with 5 mM dithiothreitol and 10 mM iodoacetamide, respectively, proteins were digested overnight with trypsin 1/25 (w/w). Vacuum dried peptides were re-suspended in 15 µl 0.1% FA (solvent A). One third of each sample was injected on a NanoLC-2DPlus system (nanoFlex ChiP module; Eksigent, ABSciex, Concord, Ontario, Canada) coupled to a TripleTOF 5600 mass spectrometer (ABSciex) operating in positive mode. Peptides were loaded on C18 columns (ChIP C-18 precolumn 300 µm ID × 5 mm ChromXP and ChIP C-18 analytical column 75 µm ID × 15 cm ChromXP; Eksigent) and were eluted using a 5%–40% gradient of solvent B (0.1% FA in Acetonitrile) for 60 minutes at a 300 nl/min flow rate. The TripleTOF 5600 was operated in high-sensitivity data-dependant acquisition mode with Analyst software (v1.6, ABSciex) on a 350–1250 m/z range. Up to 20 of the most intense multiply-charged ions (2+ to 5+) were selected for CID fragmentation, with a cycle time of 3.3s (TOP 20 discovery mode).

For protein identification, raw data were converted to Mascot Generic File format (mgf) and searched against a TAIR 10 database supplemented with a decoy database build from reverse sequences. Data were analyzed using Mascot algorithm version 2.2 (Matrix Science, UK) through ProteinScape 3.1 software (Bruker). Search parameters allowed N-acetylation (protein N-terminal), carbamidomethylation (C) and oxidation (M) as variable peptide modifications. Mass tolerances in MS and MS/MS were set to 20ppm and 0.5Da, respectively. 2 trypsin mis-cleavages were allowed. Peptide identifications obtained from Mascot were validated with a FDR <1%. A second algorithm, PEAKS DB (version 5.3, BSI Informatics) was used with the same search parameters to strengthen the identifications. Identified proteins were assessed by the total number of fragmented spectra per protein (spectral count).

Data of three (RRP41, MTR4) or two (HEN2 50 mM NaCl, HEN2 150 mM) replicates were crossed. Protein partners were considered only if present in all co-IP replicates. All proteins observed in the corresponding control replicates, including same-set and sub-set proteins, were discarded. A second filter was set using controls from 15 independent other IP experiments in *A. thaliana* carried out by other laboratories in the same MS facility. All proteins observed in any of these negative controls were discarded from the final lists of partner proteins.

Go-term analysis of proteins co-purified with MTR4-GFP was performed with DAVID (http://david.abcc.ncifcrf.gov) [71], [72].

### qRT-PCR analysis

Plants were grown on MS agar plates supplemented with 0.5% sucrose. Plates for induction of *RRP41* RNAi contained 8 µM 17β-estradiol [5]. For each target, at least three biological replicates from WT, *mtr4-1*, *mtr4-2*, *hen2-2*, *hen2-4*, *RRP41* non-induced (*RRP41* Ctrl) and *RRP41* induced (*RRP41* RNAi) were analyzed. Total RNA was isolated from 7 day-old seedlings using TRI-reagent (MRC). cDNA was synthesized from 5 µg of total RNA with SuperScript III reverse transcriptase (Invitrogen) using 37.5 pmol of oligo(dT) per 20 µl reaction according to the manufacturer's instructions. Samples were analyzed as technical triplicates in a LightCycler 480 Real-Time PCR System (Roche). Each qRT-PCR reaction contained 1× LightCycler 480 SYBR Green I Master Mix (Roche), 5 pmol of each primer, 0.5 µl of cDNA in a volume of 10 µl. *ACT2*, *TIP41* and *EXP* were used as reference mRNAs.

### Microarray analysis

Wild type, *hen2* and *mtr4* plants were grown on MS agar supplemented with 0.5% sucrose. Total RNA was extracted from two biological replicates using Nucleospin RNA plant columns (Machery & Nagel). cDNA synthesis and labeling with Cy3-dUTP or Cy5-dUTP (Perkin-Elmer-NEN Life Science Products) for fluorochrome reversal was performed as described previously [73]. Samples were hybridized to NimbleGen whole genome microrrays (Expression Omnibus (http://www.ncbi.nlm.nih.gov/geo/), accession no. GPL17057 and GPL11005) as described in [73]. Topological positions and nucleotide sequence of the 1,434,492 strand-specific NimbleGen probes are available in the FLAGdb^++^ database at http://urgv.evry.inra.fr/FLAGdb++ [45]. Two micron scanning was performed with an InnoScan900 scanner and raw data were extracted using Mapix software (Innopsys).

### Statistical analysis

Probes that mapped to repetitive sequences of the *Arabidopsis* genome were excluded from the analysis. Statistical analyses were performed with the software R. For each experiment, the raw data comprised the logarithm of median feature pixel intensities at wavelengths 635 nm (red) and 532 nm (green), respectively. The dye bias was corrected by a global intensity-dependent normalization for each chromosome and each array using the loess procedure [74] and then averaged over the technical replicates. The outputs of this procedure are two normalized intensity values per probe, one for each of the co-hybridized samples. Normalized raw data were deposited at Gene Expression Omnibus (http://www.ncbi.nlm.nih.gov/geo/), accession no. GSE48178, and at CATdb (http://urgv.evry.inra.fr/CATdb/), accession no. TIL-Ath-2011_3.

For the statistical analysis, each mutant sample was compared to the co-hybridized wild type sample. Analyses were performed for each biological replicate and independently for each chromosome and each strand (2 mutants×2 biological replicates×2 strands×5 chromosomes = 40 analysis were performed). To determine probes that behave differently between a mutant and a wild type sample, we recast the question as an unsupervised classification problem for each biological replicate, for each chromosome and for each strand. A Hidden Markov Model was developed to model the joint distribution of the two normalized hybridization intensities in order to distinguish four different biologically interpretable clusters of probes: one cluster with similar behavior in both samples (identically expressed), one cluster with higher intensity in the first sample (over-expressed), a symmetric cluster with lower intensity in the first sample (under-expressed) and one cluster with low intensities in both samples (noise) corresponding to the non-transcribed probes. The emission distribution of the noise cluster was modeled by a spherical Gaussian. A Gaussian mixture, components of which were forced to be colinear along the main axis of the ellipse representing each cluster, modeled each of the three other clusters. Data projection on the main axes allowed us to work with unidimensional mixtures and to put a unique Gaussian distribution along the associated perpendicular axis for all components. Model parameters were estimated using an adapted version of the EM algorithm taking the model constraints and the spatial dependency between probes into account. A detailed documentation of the statistical analysis can be found in dataset S2.

Probe classification into the four clusters was based on the conditional probabilities: a probe was assigned in the cluster for which the conditional probability was the highest (MAP rule). A probe was declared over-expressed in the mutant if this assignment was observed in the two biological replicates. 1860 probes were identified as overexpressed in *hen2* mutants, and 499 probes were assigned as overexpressed in *mtr4* mutants. For the majority of the estimated models, the main axis of the ellipse representing the cluster of probes under-expressed in the mutant was very closed to the main axis of the cluster with identically expressed probes. As a consequence, only a small number of probes were assigned under-expressed. Intersection of the lists with under-expressed probes across the two biological replicates revealed that no probes were underexpressed in both replicates of *hen2* or *mtr4*, respectively.

### Bioinformatics analysis

A file allowing the visualization of the upregulated probes aligned to the *Arabidopsis* genome using seqmonk software (http://www.bioinformatics.babraham.ac.uk/projects/seqmonk/) can be found in dataset S1. Probes with a unique localisation in the genome and for which a significant over-expression was detected by the statistical analysis were sorted by genome coordinates to identify upregulated regions. Only regions with at least two consecutive probes were allowed. Bioinformatic analysis was performed with adapted perl scripts. Sequences of upregulated regions were annotated using TAIR10 genome database, FLAGdb^++^, and recent studies that identified snoRNA, miRNA genes and linc RNA genes [45]–[47], [75]. Sequence alignments were performed with BLASTn and sim4 tools (for annotation of spliced transcripts) [76], [77]. Only hits with 100% identity were considered. Annotation of upregulated regions was manually curated to remove double assignments (e.g. a region that matches to snoRNA genes located in an intron of a protein coding genes was assigned as snoRNA and removed from the list of introns). For comparison with a previously published list of exosome substrates, we compared the genome coordinates of upregulated regions with coordinates of upregulated regions extracted from [5], taking the difference between different versions of TAIR into account.

### Accession numbers (Arabidopsis Genome Initiative, AGI)

*ATMTR4*: At1g59760; *HEN2:* At2g06990; *RRP41*: At3g61620.

## Supporting Information

Dataset S1 Seqmonk file to visualize the location of significantly upregulated probes in the Arabidopsis genome, and instructions for opening the file using the free seqmonk software.
(ZIP)

Dataset S2 Detailed documentation of the statistical analysis of the tiling data.
(PDF)

Figure S1 Plant MTR4 proteins have an insertion in the inner loop of the arch domain. Top: Sequences of MTR4 and HEN2 proteins from selected plant species were aligned to the sequence of *S. cerevisiae* MTR4 (highlighted in orange). The alignment is shown for 50 aminoacids of the arch domain. Athaliana, *Arabidopsis thaliana* (thale cress); Thalophila, *Thelluniella halophila* (Salt cress); Sitalica, *Setaria italica* (Foxtail millet); Mguttatus, *Mimulus guttatus* (Monkey flower); Pvulgaris, *Phaesolus vulgaris* (Common bean); Mtrunculata, *Medicago trunculata* (Barrel medic); Mesculenta, *Manihot esculenta* (Cassava); Ptrichocarpa, *Populus trichocarpa* (Poplar); Csativus, *Cucumis sativus* (cucumber); Egrandis, *Eucalyptus grandis* (Eucalyptus); Smoellendorfii, *Selaginella moellendorfii* (Spikemoss); Ppatens, *Physcomitrella patens* (Moss); Scerevisiae, *Saccharomyces cerevisiae* (Bakers yeast). Left: Model of the arch domains of AtMTR4 and HEN2. AtMTR4 and HEN2 structures were modeled using the yeast MTR4 structure as template. Only the arch domain is shown (from the top). Yeast MTR4 in orange, HEN2 in green, AtMTR4 in blue. A dashed red line indicates the insertion of 9 amino acids present in AtMTR4.
(PDF)

Figure S2 Full sequence alignment of MTR4 and HEN2 proteins from selected species. Amino acids are colored by the ClustalX color scheme. Boxes below the alignment illustrate RecA domains (blue), the winged helix domains (yellow), the arch domain (red, the black box depicts the KOW domain) and the C-terminal bundle domain (pink). Characteristic differences between plant MTR4 and HEN2 sequences are marked by stars. Athaliana, *Arabidopsis thaliana* (thale cress); Thalophila, *Thelluniella halophila* (Salt cress); Sitalica, *Setaria italica* (Foxtail millet); Mguttatus, *Mimulus guttatus* (Monkey flower); Pvulgaris, *Phaesolus vulgaris* (Common bean); Mtrunculata, *Medicago trunculata* (Barrel medic); Mesculenta, *Manihot esculenta* (Cassava); Ptrichocarpa, *Populus trichocarpa* (Poplar); Csativus, *Cucumis sativus* (cucumber); Egrandis, *Eucalyptus grandis* (Eucalyptus); Smoellendorfii, *Selaginella moellendorfii* (Spikemoss); Ppatens, *Physcomitrella patens* (Moss).
(PDF)

Figure S3 MTR4-GFP co-localises with nucleolar marker proteins. Transient expression of fluorescent fusion proteins in *Nicotiana benthamiana* leaves. MTR4-GFP is shown in green, RFP-fusion proteins are shown in red. Fibrillarin-RFP and XRN2-RFP are known nucleolar markers; SRP34a-RFP was used as a nucleoplasmic marker. The phase contrast picture is shown on the right. Scale bars: 15 µm.
(PDF)

Figure S4 HEN2-GFP co-localises with a nucleoplasmic marker protein. Transient expression of fluorescent fusion proteins in *Nicotiana benthamiana* leaves. HEN2-GFP is shown in green, RFP-fusion proteins are shown in red. Fibrillarin-RFP and XRN2-RFP were used as nucleolar markers; SRP34a-RFP was used as a nucleoplasmic marker. The phase contrast picture is shown on the right. Scale bars: 15 µm.
(PDF)

Figure S5 MTR4 and HEN2 have distinct localization patterns. The distribution of the indicated GFP-fusion proteins in root cells of stable *Arabidopsis* transformants is shown on the left. The middle column shows DAPI staining. Please note that take-up of DAPI by intact, living plant tissue is slow and can lead to a strong background signal from cell walls. No, Nucleolus; Np, Nucleoplasm; Cp, Cytoplasm. Scale bars: 5 µm.
(PDF)

Figure S6 HEN2-GFP is localized in nucleoplasmic foci. Co-expression of HEN2-GFP and the nucleoplasmic marker protein SRP34a in leaves of stable *Arabidopsis* transformants. Nucleoplasmic foci were observed in all cell types of all stable transformants. Scale bars: 15 µm.
(PDF)

Figure S7 Sequence alignment of human RBM7 and *Arabidopsis* At4g10110.
(PDF)

Figure S8 Supplemental information about selected known exosome substrates from Fig. 4.
(DOCX)

Figure S9 Accumulation of unspliced transcripts in *hen2* mutants. qRT-PCR. A Diagram of the genomic locus indicated by the respective AGI number is shown at the top of each panel. Annotated mRNA genes are represented as arrows with dark blue boxes for the CDS, light blue boxes for 3′ and 5‚ UTRs, and a light blue line for introns. Red bars above the diagram represent probes detected in the microarray analysis. Green arrows above or below the diagram depict the location of qRT-PCR primers. The corresponding qRT-PCR results for each primer pair are given as fold-change relative to WT in the histograms below each diagram. *mtr4-1* in red, *mtr4-2* in orange, *hen2-2* in light green, *hen2-4* in dark green, *RRP41* control in light grey, *RRP41* RNAi in dark grey. Error bars = SD in three biological replicates. **A.** At3g26510 (with 4 predicted splice variants). qRT-PCR results suggest that *hen2* and *RRP41* RNAi plants accumulate a population of transcripts some of which still contain the unspliced acceptor site of the first intron (panel V114a/b) and some of which still contain the 2nd intron (panel V112a/b). **B.** At1g58602. Transcripts comprising the unspliced 2nd exon/intron donor site accumulate in *hen2* and *RRP41* RNAi plants. **C.** At3g43160. Both spliced and unspliced transcripts corresponding to the 3′ region of the At3g43160 locus accumulate in *hen2* and *RRP41* RNAi plants.
(PDF)

Figure S10 Unspliced transcripts from the At1g79270 locus are polyadenylated. Sequences of 3′ RACE PCR products obtained from *hen2-4* samples. cDNA synthesis was initiated using oligo-dT as primer. 3′ RACE PCR was performed with V113a (green arrow) as forward primer, and the adapter sequence of the oligo-dT primer as a reverse primer. PCR products obtained from *hen2-4* samples were cloned and sequenced. The genomic sequence is given above the line, with intronic sequence in purple. Red arrows mark donor and acceptor splice sites. Non-encoded nucleotides are in green.
(PDF)

Figure S11 Upregulation of mRNAs in *hen2* mutants. Accumulation of mRNAs was tested by qRT-PCR using primer pairs located in 5′, central or 3′ regions of the annotated transcripts as indicated below each panel. *mtr4-1* in red, *mtr4-2* in orange, *hen2-2* in light green, *hen2-4* in dark green, *RRP41* control in light grey, *RRP41* RNAi in dark grey. Error bars = SD in three biological replicates.
(PDF)

Figure S12 mRNAs are not systematically detected in all replicates. Accumulation of mRNAs was tested by qRT-PCR using primer pairs located in 5′ or 3′ regions of the annotated transcripts as indicated below each panel. Panels on the left show the qRT-PCR results for exactly the same samples that have been used for hybridisation to the tiling arrays. Panels on the right show the results obtained in 3 independent replicates grown in the same culture conditions. A possible explanation for the inconsistence between the replicates could be that mRNAs are not bona-fide substrates of exosome-mediated RNA degradation and might rather be upregulated due to indirect effects. Other types of transcripts such as short mRNA-derived regions, introns, unspliced transcripts or several types of non- coding RNAs are consistently observed in all replicates. *mtr4-1* in red, *mtr4-2* in orange, *hen2-2* in light green, *hen2-4* in dark green, *RRP41* control in light grey, *RRP41* RNAi in dark grey. Error bars = SD in three biological replicates.
(PDF)

Figure S13 Loss of HEN2 or the exosome is associated with increased levels of polyadenylated snoRNA precursors. Oligo-dT primed cDNA was used for 3′ RACE- PCR, with primer E5 and the adapter sequence of the cDNA synthesis primer as forward and reverse primers, respectively. PCR products were separated on 2% agarose gels, transferred to Hybond XL membranes, and hybridized with radiolabeled probes E6 (mid panel) and E8 (lower panel). The diagram below illustrates location of primers and probes with respect to the snoRNA genes in this region (see also Fig. 9).
(PDF)

Figure S14 Polyadenylated transcripts partially antisense to AT5G44306. Sequences were amplified by 3′ RACE from oligo-dT primed cDNA from the indicated samples using a primer (indicated by the purple arrow) situated antisense to the 5′ region of AT5G44306. Non-encoded nucleotides are in green.
(PDF)

Figure S15 *mtr4* mutants accumulate exosome substrates to lower levels than *hen2* mutants. Boxplot showing averaged intensity values for the overexpressed probes identified in each comparison of the tiling array analysis. The first two rows show the intensity values in WT and *hen2* samples for all probes overexpressed in both biological replicates of *hen2*. The third and fourth rows show the intensity values in WT and *mtr4* samples for all probes overexpressed in both biological replicates of *mtr4*. The averaged intensities in *mtr4* samples are significant lower than the averaged intensities in *hen2* samples (p-value<1e -3). The fifth and sixth rows show the intensity values in *hen2* and *mtr4* for the common probes (overexpressed in both mutants). Again, the mean average intensity in *mtr4* samples is lower than the mean average intensity in *hen2* samples (p-value<1e -3).
(PDF)

Figure S16 The microarray analysis probably underestimates the contribution of HEN2 to nuclear RNA surveillance. A diagram of the genomic locus indicated by the respective AGI number is shown at the top of each panel. Annotated mRNA genes are represented as arrows with dark blue boxes for the CDS, light blue boxes for 3′ and 5′ UTRs, and a light blue line for introns. Red bars above the diagram represent probes detected in the microarray analysis. Green arrows above or below the diagram depict the location of qRT-PCR primers. The corresponding qRT-PCR results for each primer pair are given as fold-change relative to WT in the histograms below each diagram. *mtr4-1* in red, *mtr4-2* in orange, *hen2-2* in light green, *hen2-4* in dark green, *RRP41* control in light grey, *RRP41* RNAi in dark grey. Error bars = SD in three biological replicates. Upper panel: The upregulation of two stretches in the 5′ region of At1g20100 in *RRP41* RNAi lines (indicated by the red double arrows) was detected in a previous tiling microarray study [5]. Our microarray array detected only a portion of this region (indicated by the red bars above the diagram). However, we could confirm the upregulation of the uppermost 600 kb of At1g20100 in both *hen2* alleles by qRT-PCR. Middle panel: A portion of the fifth intron of At5g27720 was previously identified as a target of the exosome core complex [5] (indicated by the red double arrows). In our microarray analysis, only one probe in this region was declared statistically significant. Since we considered only regions with at least two consecutive probes, this region was omitted from the data interpretation. Nevertheless, qRT-PCR data show that a portion of the fifth intron is upregulated in *hen2* mutants, while levels of pre-mRNA or mature mRNAs are similar to WT. The presence of polyadenylated transcripts corresponding either to the entire intron or to shorter degradation intermediates in *hen2* and *RRP41* RNAi samples was further confirmed by cloning of 3′ RACE products (not shown). Lower panel: A previous tiling study [5] identified a short stretch derived from the 5′ region of At4g02890 as a target of the exosome core complex (indicated by the red double arrows). This region was not detected in our tiling analysis, but its upregulation was easily detected by qRT-PCR. The discrepancy between the two tiling studies is probably largely explained by the different arrays designs (the NimbleGen 732K array is not designed to detect very small regions) and by different statistical analysis procedures. Moreover, the accumulation of exosome targets may also vary with growth conditions. In fact, several of our validated targets have not been picked up in the previous genome-wide analysis [5], indicating that both tiling studies under-estimate the contribution of exosome-mediated RNA surveillance.
(PDF)

Figure S17 A fraction of AtMTR4-GFP can be detected in the nucleoplasm. Intracellular distribution of AtMTR4-GFP in root cells of a stable *Arabidopsis* transformant. The nucleoplasmic fraction of AtMTR4-GFP (white arrows) is more visible in individual transformants displaying relative weak transgene expression, which is not representative for the majority of the investigated plant lines.
(PDF)

Table S1 Mass-spectrometric analysis of RRP41 IP experiments: list of peptides.
(XLSX)

Table S2 Mass- spectrometric analysis of MTR4 IP experiments: list of peptides.
(XLSX)

Table S3 Mass- spectrometric analysis of HEN2 IP experiments: list of peptides.
(XLSX)

Table S4 Analysis of microarray data. Regions corresponding to short stretches of mRNAs, unspliced transcripts, and 3′ or 5′ extended mRNAs.
(XLSX)

Table S5 Analysis of microarray data. Regions corresponding to introns.
(XLSX)

Table S6 Analysis of microarray data. Regions corresponding to mRNAs.
(XLSX)

Table S7 Analysis of microarray data. Regions corresponding to transposable elements.
(XLSX)

Table S8 Analysis of microarray data. Regions corresponding to snoRNA precursors.
(XLSX)

Table S9 Analysis of microarray data. Regions corresponding to linc and other RNAs.
(XLSX)

Table S10 Analysis of microarray data. Potential antisense transcripts.
(XLSX)

Table S11 Analysis of microarray data. Non-annotated regions.
(XLSX)

Table S12 Gene descriptions and biological functions for the mRNAs upregulated in hen2 and/or mtr4 samples.
(XLSX)

Table S13 Primers used in this study.
(XLSX)
