# Supplementary material for: The RNA Helicases AtMTR4 and HEN2 Target Specific Subsets of Nuclear Transcripts for Degradation by the Nuclear Exosome in Arabidopsis thaliana
Source: PLoS Genet. 2014 Aug 21;10(8):e1004564. doi: 10.1371/journal.pgen.1004564 (PMC4140647; doi:10.1371/journal.pgen.1004564)
Supplement: Dataset S2 — Detailed documentation of the statistical analysis of the tiling data. (PDF) [file pgen.1004564.s002.pdf]

HMM with mixture as emission distribution for tiling array data

An Hidden Markov Model is proposed to model the joint distribution of two hybridization intensities, taking into account the spatial dependence of the probes along the genome. In this study, it is developed for a constant number of clusters fixed at $K = 4$, where these four clusters are biologically interpretable (presented Figure 1): the noise group, the identical group and the two differentially expressed groups. To simplify the notation, we note 0 for the noise group, 1 for the identical group and 2 and 3 for the differentially expressed groups. The idea of this model is already published in [1] but instead of modeling each distribution emission by a Gaussian distribution, a mixture of distributions is considered for each emission distribution. It leads to a more flexible modeling, better adapted to fit the data than an HMM with classical parametric distributions to model the emission distributions.

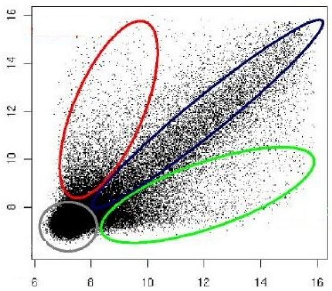

Figure 1: Schematic explanation of the 4 groups to consider when comparing two samples. Example of transcriptomic data.

Model

We assume that the observed data $X = \{X_1, ..., X_n\}$ result from a HMM, with $X_t = (X_{1t}, X_{2t})$ the log-intensities of probe t in the two conditions. The hidden path $\{Z_t\}$ is a 4-state homogeneous Markov chain with transition matrix Π and stationnary distribution m. The observations $\{X_t\}$ are independent conditionally to Z. To make the emission distributions more flexible than in the classical setting, each emission distribution is assumed to be itself a mixture of parametric distributions:

$$(X_t|Z_t = k) \sim \sum_{\ell=1}^{L_k} \eta_{k\ell} f(.;\theta_{k\ell}) \ , \quad \text{for } k = 1, ..., 4$$

where L_k is the component number in the mixture for the k-th emission distribution and $\eta_{k\ell}$ is the mixing proportion of the ℓ-th component for the cluster k ($\forall \ell \in \{1, ..., L_k\}$, $0 < \eta_{k\ell} < 1$ and $\sum_\ell \eta_{k\ell} = 1$). We denote by L the total number of components of the model: $\sum_{k=1}^{K} L_k = L$ and by $\Theta = (\Pi, m, \{\eta_{k\ell}\}_{k,\ell}, \{\theta_{k\ell}\}_{k,\ell})$ the vector of model parameters.

This mixture of bidimensional Gaussian distributions can be recast as an unidimensional mixture by considering three axes Δ_1, Δ_2 and Δ_3, concurrent at the barycentre of the noise group, corresponding respectively to the main axis of the ellipse representing groups 1, 2 and 3. The Gaussian components of the k-th cluster are then colinear along the axis Δ_k (see Figure 2) leading to a more tractable model (see 2).

The details of the model are the following:

- The noise group is a special group, considered as circular and modeled by a spherical Gaussian :

$$(X_t|Z_t = 0) \sim \mathcal{N}\left(\begin{pmatrix}\mu_0^1\\ \mu_0^2\end{pmatrix}, \sigma^2 I_2\right) \ .$$

- Each of the three other groups is modeled by a Gaussian mixture. Let (U_{tk}, V_{tk}) be the coordinates of (X_{1t}, X_{2t}) in the orthonormal basis $(\Delta_k, \Delta_k^\perp)$.

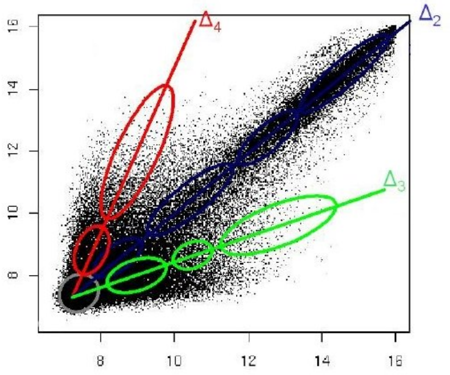

Figure 2: Schematic representation of Gaussian mixtures in each cluster, along the three axes.

We consider an unidimensional Gaussian mixture along each axis Δ_k and a unique distribution for all components along $\Delta_k^{\perp}$:

$$(V_{tk}|Z_t = k) \sim \mathcal{N}(0, \sigma^2) \text{ and } (U_{tk}|Z_t = k) \sim \psi_k = \sum_{\ell=1}^{L_k} \eta_{k\ell} f(.;\theta_{k\ell}) \ ,$$

with $\eta_{k\ell}$ is the proportion of the ℓ-th component for the cluster k and $f(.;\theta_{k\ell}) \sim \mathcal{N}(\mu_{kl}, \sigma_{kl}^2)$.

Inference

The standard strategy for maximum likelihood inference of HMM relies on the Expectation-Maximisation (EM) algorithm ([2]). The E-step aims at calculating the conditional distribution of the hidden path given the observed data, with the current value of the parameters of the model.

This can be achieved via the forward-backward algorithm (see [3] for further details). At iteration h, it only requires the current estimate of the transition matrix Π^h and the current estimates of the emission densities at each observation point: $\psi_k^h(X_t)$. At the M-step, the parameters estimates are obtained by maximizing $\mathbb{E}\left[\log P(X, Z; \Theta)|X\right]$ in Θ. We have:

$$\mathbb{E}\left[\log P(X, Z; \Theta)|X\right] \quad = \quad \mathbb{E}\left[\log P(Z; \Pi, m)|X\right] + \mathbb{E}\left[\log P(X|Z; \eta, \theta)|X\right] \ .$$

The maximization of the first term is straightforward and results in the estimation of Π^{h+1} and m^{h+1}. As the emission distributions are inherently mixture, the maximisation of the second term requires some specific development. By definition we have:

$$\mathbb{E}\left[\log P(X|Z; \eta, \theta)|X\right] = \sum_{t=1}^{n}\sum_{k=1}^{K} \tau_{tk} \log\left[\sum_{\ell=1}^{L_k} \eta_{k\ell} f(X_t; \theta_{k\ell})\right] \ ,$$

where τ_{tk} is the posterior probability for an observation t to belong to the cluster k, defined by $P(Z_t = k|X)$. Noting that the term corresponding to cluster k is a weighted version of the likelihood of an independent mixture, we introduce a second hidden variable $\{W_t\}_t$ which refers to component ℓ within cluster k. As a consequence, $\mathbb{E}\left[\log P(X|Z; \Theta)|X\right]$ can be rewritten as:

$$\mathbb{E}\left[\log P(X, W|Z; \Theta)|X\right] - \mathbb{E}\left[\log P(W|X, Z; \Theta)|X\right]. \qquad (1)$$

Similarly to the classical EM algorithm with only one latent variable, the fundamental property established by [2] can be applied to Equation (1): maximising $\mathbb{E}\left[\log P(X|Z; \Theta)|X\right]$ amounts to only maximising the first term, which is equal to:

$$\mathbb{E}\left[\log P(X|W,Z;\Theta)|X\right] + \mathbb{E}\left[\log P(W|Z;\Theta)|X\right] = \sum_t \tau_{tk} \sum_\ell \delta_{tk\ell} \log f(X_t;\theta_{k\ell}) + \sum_t \tau_{tk} \sum_\ell \delta_{tk\ell} \log \eta_{k\ell}$$

where

$$\delta_{tk\ell} = \mathbb{E}\left[W_{tk\ell} = 1 | Z_t = k, X_t\right].$$

We now face the inference of a standard parametric mixture, for which we know the solution. At the E-step we have:

$$\widehat{\delta}_{tk\ell} = \frac{\widehat{\eta}_{k\ell} f(X_t;\widehat{\theta}_{k\ell})}{\sum_{\ell=1}^{L_k} \widehat{\eta}_{k\ell} f(X_t;\widehat{\theta}_{k\ell})},$$

and for the M-step:

$$\widehat{\eta}_{k\ell} = \frac{\sum_t \widehat{\tau}_{tk} \widehat{\delta}_{tk\ell}}{\sum_t \widehat{\tau}_{tk}} ,$$

and

$$\widehat{\theta}_{k\ell} = \underset{\theta_{k\ell}}{\operatorname{argmax}} \sum_t \widehat{\tau}_{tk} \sum_\ell \widehat{\delta}_{tk\ell} \log f(X_t;\theta_{k\ell}) .$$

Once the parameters estimation is done, we get for each probe the posterior probability τ_{tk} to belong to cluster k. The classification of probes is then performed according to the *Maximum A Posteriori* rule which consists in assigning a probe in the cluster for which the posterior probability is the highest.

References

[1] C. Bérard, M.L. Martin-Magniette, V. Brunaud, S. Aubourg and S. Robin. Unsupervised Classification for Tiling Arrays: ChIP-chip and Transcriptome. *Statistical Applications in Genetics and Molecular Biology* **10**:1, Article 1, 2011.

[2] A.P. Dempster, N.M. Laird and D.B. Rubin. Maximum likelihood from incomplete data via the EM algorihtm (with discussion). *J. R. Statis. Soc. B* **39** 1-38, 1977.

[3] L.R. Rabiner. A tutorial on Hidden Markov Models and selected applications in Speech Recognition. *Proceedings of the IEEE*, 1989.
