# Supplementary material for: The RNA Helicases AtMTR4 and HEN2 Target Specific Subsets of Nuclear Transcripts for Degradation by the Nuclear Exosome in Arabidopsis thaliana
Source: PLoS Genet. 2014 Aug 21;10(8):e1004564. doi: 10.1371/journal.pgen.1004564 (PMC4140647; doi:10.1371/journal.pgen.1004564)

MTR4_Athaliana	583	Y C L P F L L P N R A V C L D C T N D - - D E E P Q S F S I E D Q D T W G V I M K F N K V K S L S E	630
MTR4_Thalophila	585	Y C L P F L L P N R A V C L D C T N D - - D G E P Q S F S I E N Q D A W G V I M K F S K V K S T S E	632
MTR4_Sitalica	597	H V L P F L Q P G R L V R L E Y S - - - - T D E P A T F S I D E N I T W G I I I N F E K V K S H G E	642
MTR4_Mguttatus	589	H C L P F L Q P G R L V S I Q C T K N - - D E D S S S F S M K D E I T W G V I I N F E R V K T V S E	636
MTR4_Pvulgaris	582	H C L P F L Q P G R L V S L E C T S S - - N E D L T P I F I E D Q L T W G L V V N F E R V K S V S D	629
MTR4_Mtruncatula	584	H C L P Y L Q P G R L V S L Q C T S S - - E E D L V P I F I E D Q L T W G L I I N F E R I K G V S E	631
MTR4_Mesculenta	586	H C L P F L Q A G R I V C L Q C T G T - - D E N S P S F S I E D Q V T W G V I I N F E R V K E F S E	633
MTR4_Ptrichocarpa	607	H C L S Y L Q S G R L V C I Q C T E S - - D D K S P S F L I E D L V T W G V I V N F D R V K G V S D	654
MTR4_Csativus	588	Y C L P F L Q P G R L V S I E C N R N - - D E I S S T F S I K D Q V T W G L I I N F Q R V K G V S E	635
MTR4_Egrandis	588	Y S L P F L Q P G R L V S I E C T S S - - D K S G S S F S M E D Q A T W G V I I N F E R V R S A S E	635
MTR4_Smoellindorffii	578	S C L P F L Q P G R L V R I F R S A D A V E Q Q N S V L M E Q D P A V W G V I I N F E K A Q - - S K	625
MTR4_Ppatens	605	Y S L P Y L Q P G R L V Q I A R A T N - - Q D D L T V P V K K V T P V W G V I V N F E K V Q T A A K	652
MTR4_Scerevisiae	704	N A L S F L Q P G R L V E I S V N G K - - - - - - - - - - - - D N Y G W G A V V D F A K R I N K R N	741
HEN2_Athaliana	601	R V L C F L D T G R L V K I R E G G T - - - - - - - - - - - - - D W G W G V V V N V V K N S S V G T	637
HEN2_Thalophila	601	R V L C F L D T G R L I K I R E G G T - - - - - - - - - - - - - D W G W G V V V N V V K K P S V G T	637
HEN2_Sitalica	609	R A L L Y L V P G R L V K V R D G S T - - - - - - - - - - - - - D W G W G V V V N V V K K P P A S G	645
HEN2_Mguttatus	598	R V L S F L Q P G R L V K V R E G G T - - - - - - - - - - - - - D W G W G V V V N V V K K P P A P S	634
HEN2_Pvulgaris	597	I I L Y F L V P G R L I K V R E G G T - - - - - - - - - - - - - D W G W G V V V N V V K K P - - - -	629
HEN2_Mtruncatula	600	M I L Y F L V P G R L I K V R E G G T - - - - - - - - - - - - - D W G W G V V V N V V K K P - - - -	632
HEN2_Mesculenta	593	R V L Y Y L C T G R L I K V R E G G T - - - - - - - - - - - - - D W G W G V V V N V V K K P A P G L	629
HEN2_Ptrichocarpa	591	R I L Y Y L C T G R L I K V R E G G T - - - - - - - - - - - - - D W G W G V V V N V V K K P T A G L	627
HEN2_Csativus	620	R V L Y F L L P G R L V K V R E G G T - - - - - - - - - - - - - D W G W G V V V N V V K K P S A G L	656
HEN2_Egrandis	599	R V L Y F L L P G R L V R I R E G G T - - - - - - - - - - - - - D W G W G V V V N V I K K P S T G L	635
HEN2_Smoellindorffii	571	R I I V F L Q P G R L V K V R D G A D - - - - - - - - - - - - - D W G W G V V I N V V K K P T P G G	607
HEN2_Ppatens	617	R V L L F M K P G R L V K I R D G S D - - - - - - - - - - - - - E W G W G V V V H V V K K P T S N Q	653

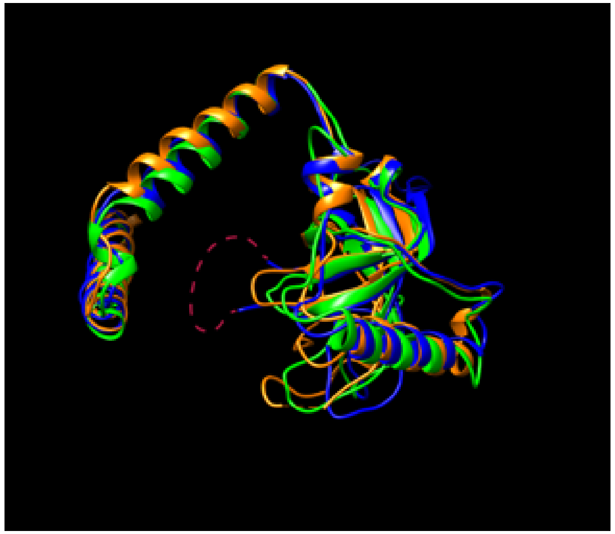

Supplement: Figure S1 — Plant MTR4 proteins have an insertion in the inner loop of the arch domain. Top: Sequences of MTR4 and HEN2 proteins from selected plant species were aligned to the sequence of S. cerevisiae MTR4 (highlighted in orange). The alignment is shown for 50 aminoacids of the arch domain. Athaliana, Arabidopsis thaliana (thale cress); Thalophila, Thelluniella halophila (Salt cress); Sitalica, Setaria italica (Foxtail millet); Mguttatus, Mimulus guttatus (Monkey flower); Pvulgaris, Phaesolus vulgaris (Common bean); Mtrunculata, Medicago trunculata (Barrel medic); Mesculenta, Manihot esculenta (Cassava); Ptrichocarpa, Populus trichocarpa (Poplar); Csativus, Cucumis sativus (cucumber); Egrandis, Eucalyptus grandis (Eucalyptus); Smoellendorfii, Selaginella moellendorfii (Spikemoss); Ppatens, Physcomitrella patens (Moss); Scerevisiae, Saccharomyces cerevisiae (Bakers yeast). Left: Model of the arch domains of AtMTR4 and HEN2. AtMTR4 and HEN2 structures were modeled using the yeast MTR4 structure as template. Only the arch domain is shown (from the top). Yeast MTR4 in orange, HEN2 in green, AtMTR4 in blue. A dashed red line indicates the insertion of 9 amino acids present in AtMTR4. (PDF) [file pgen.1004564.s003.pdf]
