# Supplementary figures and images for: The RNA Helicases AtMTR4 and HEN2 Target Specific Subsets of Nuclear Transcripts for Degradation by the Nuclear Exosome in Arabidopsis thaliana
Source: PLoS Genet. 2014 Aug 21;10(8):e1004564. doi: 10.1371/journal.pgen.1004564 (PMC4140647; doi:10.1371/journal.pgen.1004564)

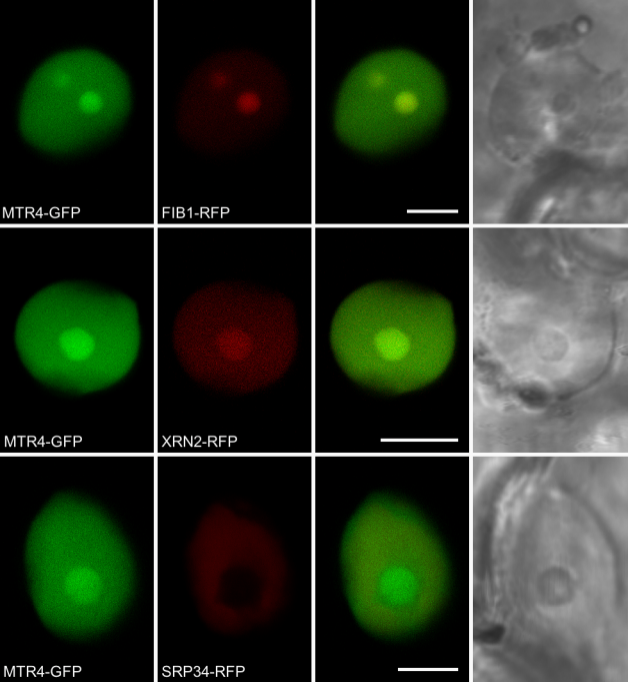

Supplement: Figure S3 — MTR4-GFP co-localises with nucleolar marker proteins. Transient expression of fluorescent fusion proteins in Nicotiana benthamiana leaves. MTR4-GFP is shown in green, RFP-fusion proteins are shown in red. Fibrillarin-RFP and XRN2-RFP are known nucleolar markers; SRP34a-RFP was used as a nucleoplasmic marker. The phase contrast picture is shown on the right. Scale bars: 15 µm. (PDF) [file pgen.1004564.s005.pdf]

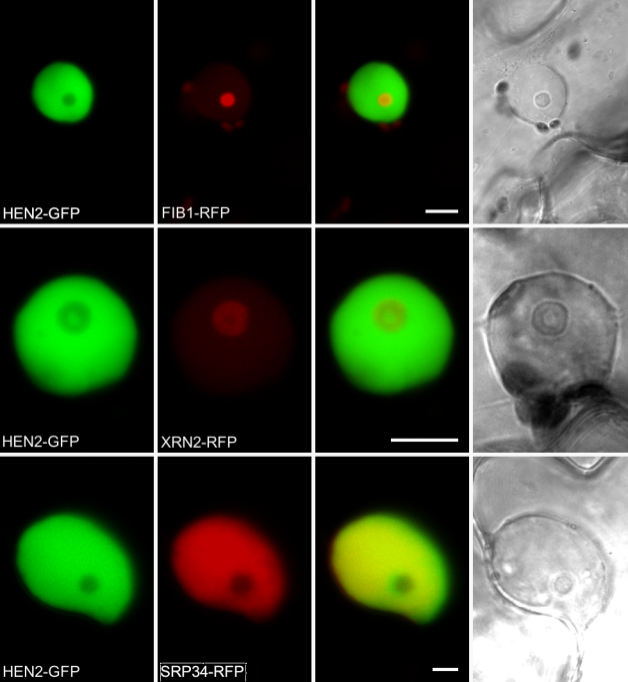

Supplement: Figure S4 — HEN2-GFP co-localises with a nucleoplasmic marker protein. Transient expression of fluorescent fusion proteins in Nicotiana benthamiana leaves. HEN2-GFP is shown in green, RFP-fusion proteins are shown in red. Fibrillarin-RFP and XRN2-RFP were used as nucleolar markers; SRP34a-RFP was used as a nucleoplasmic marker. The phase contrast picture is shown on the right. Scale bars: 15 µm. (PDF) [file pgen.1004564.s006.pdf]

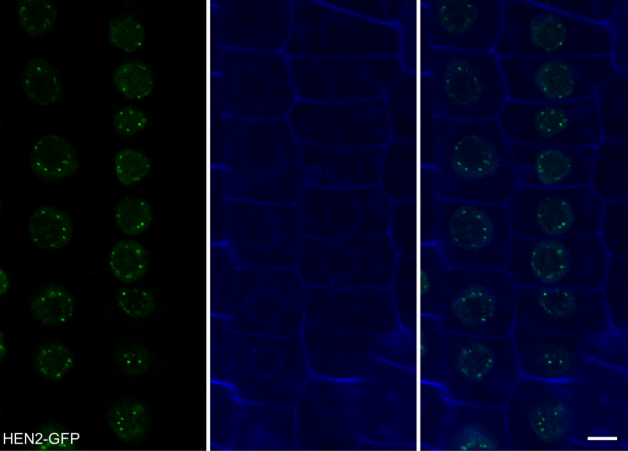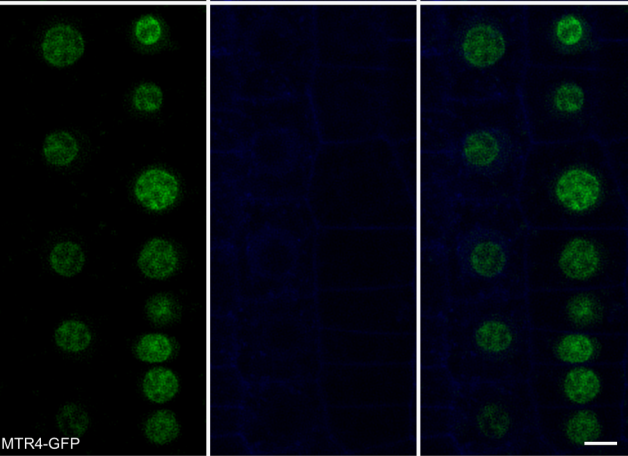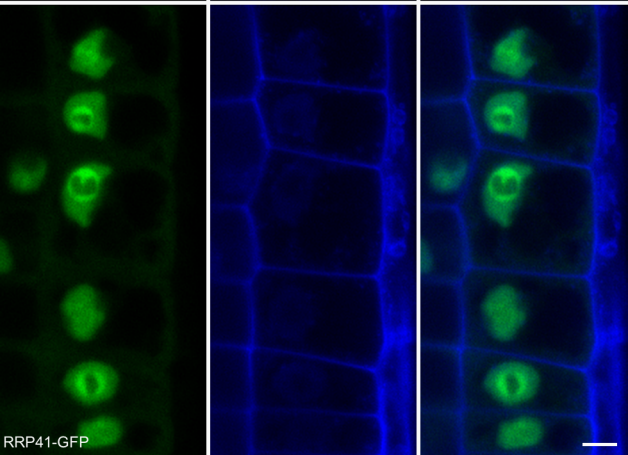

Supplement: Figure S5 — MTR4 and HEN2 have distinct localization patterns. The distribution of the indicated GFP-fusion proteins in root cells of stable Arabidopsis transformants is shown on the left. The middle column shows DAPI staining. Please note that take-up of DAPI by intact, living plant tissue is slow and can lead to a strong background signal from cell walls. No, Nucleolus; Np, Nucleoplasm; Cp, Cytoplasm. Scale bars: 5 µm. (PDF) [file pgen.1004564.s007.pdf]

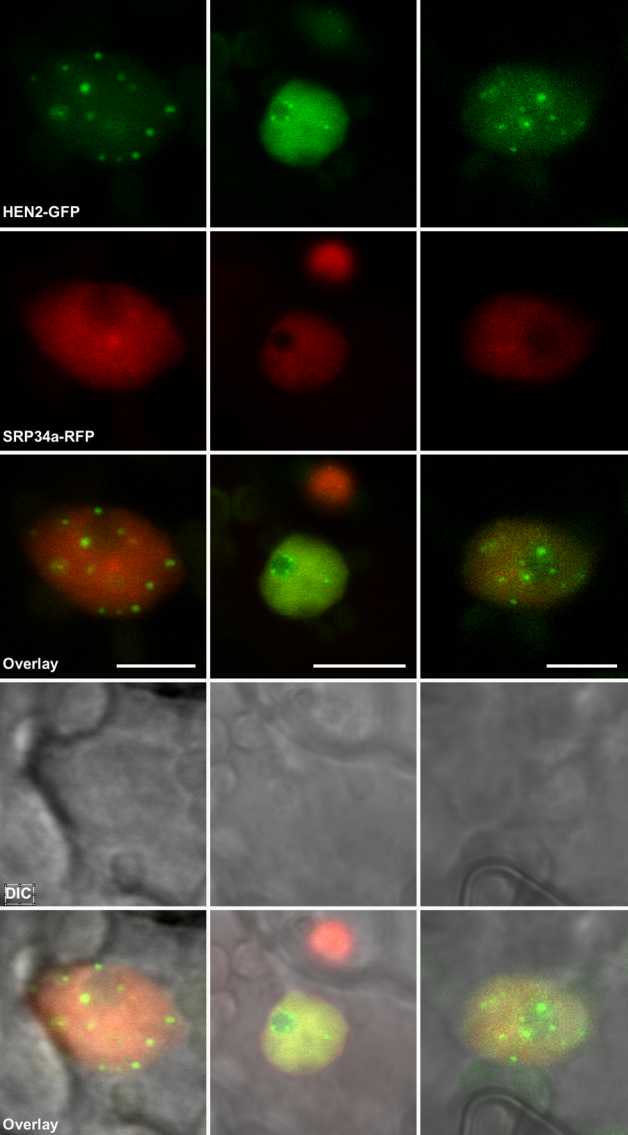

Supplement: Figure S6 — HEN2-GFP is localized in nucleoplasmic foci. Co-expression of HEN2-GFP and the nucleoplasmic marker protein SRP34a in leaves of stable Arabidopsis transformants. Nucleoplasmic foci were observed in all cell types of all stable transformants. Scale bars: 15 µm. (PDF) [file pgen.1004564.s008.pdf]

## A At3g26510

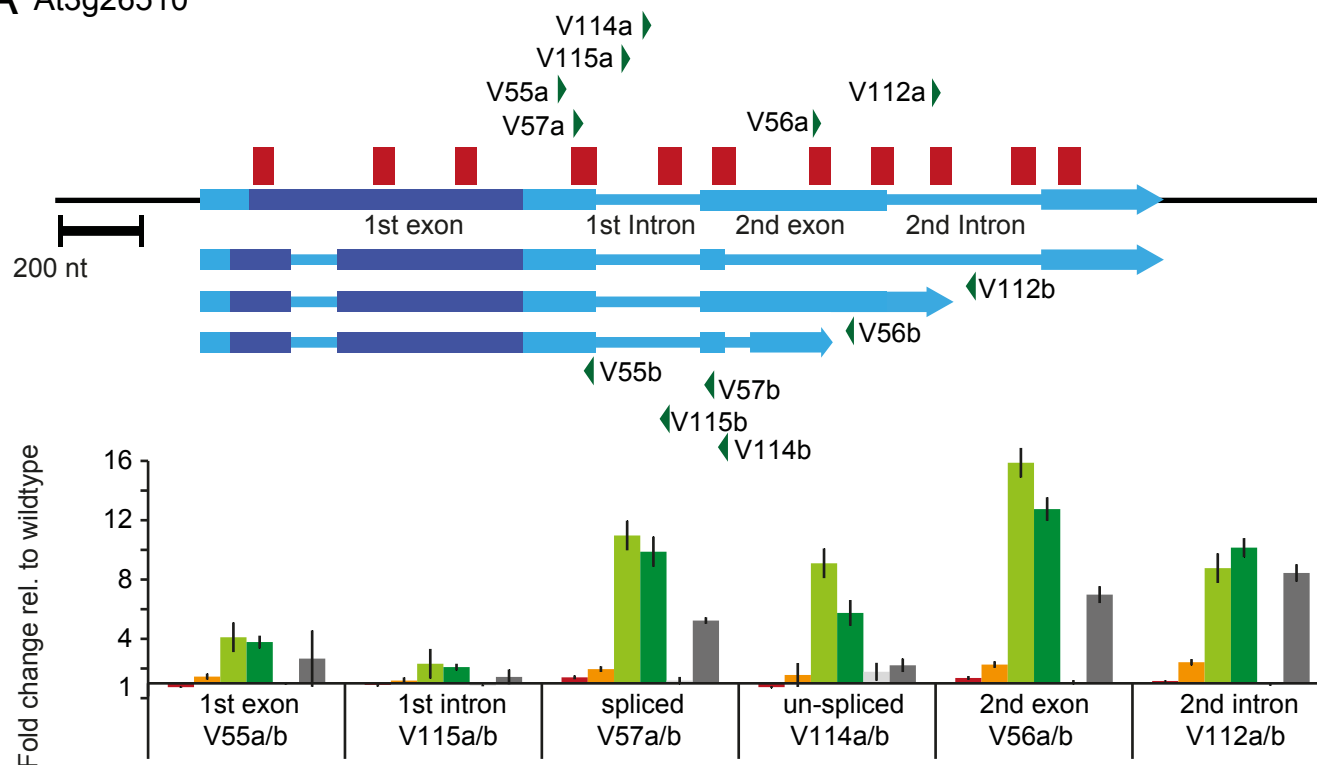

## B At1g58602

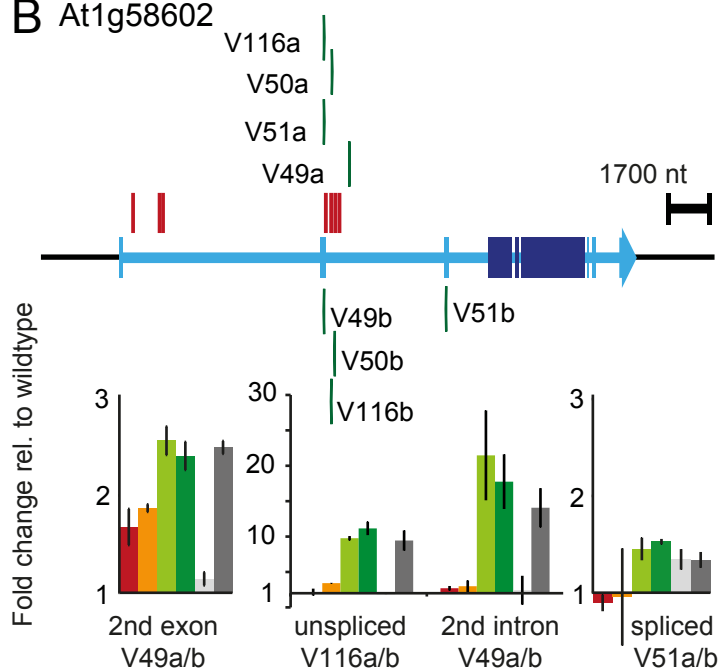

## C At3g43160

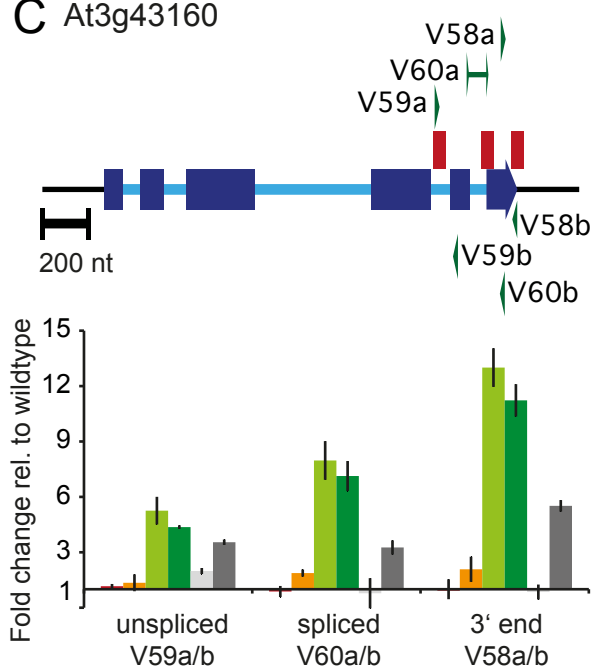

■ *mtr4-1*
■ *hen2-2*
■ *RRP41* ctrl  
■ *mtr4-2*
■ *hen2-4*
■ *RRP41* RNAi

exon 
  CDS 
  detected probes  
 intron 
 → mRNA 
 ↗ qPCR primer

Supplement: Figure S9 — Accumulation of unspliced transcripts in hen2 mutants. qRT-PCR. A Diagram of the genomic locus indicated by the respective AGI number is shown at the top of each panel. Annotated mRNA genes are represented as arrows with dark blue boxes for the CDS, light blue boxes for 3′ and 5‚ UTRs, and a light blue line for introns. Red bars above the diagram represent probes detected in the microarray analysis. Green arrows above or below the diagram depict the location of qRT-PCR primers. The corresponding qRT-PCR results for each primer pair are given as fold-change relative to WT in the histograms below each diagram. mtr4-1 in red, mtr4-2 in orange, hen2-2 in light green, hen2-4 in dark green, RRP41 control in light grey, RRP41 RNAi in dark grey. Error bars = SD in three biological replicates. A. At3g26510 (with 4 predicted splice variants). qRT-PCR results suggest that hen2 and RRP41 RNAi plants accumulate a population of transcripts some of which still contain the unspliced acceptor site of the first intron (panel V114a/b) and some of which still contain the 2nd intron (panel V112a/b). B. At1g58602. Transcripts comprising the unspliced 2nd exon/intron donor site accumulate in hen2 and RRP41 RNAi plants. C. At3g43160. Both spliced and unspliced transcripts corresponding to the 3′ region of the At3g43160 locus accumulate in hen2 and RRP41 RNAi plants. (PDF) [file pgen.1004564.s011.pdf]

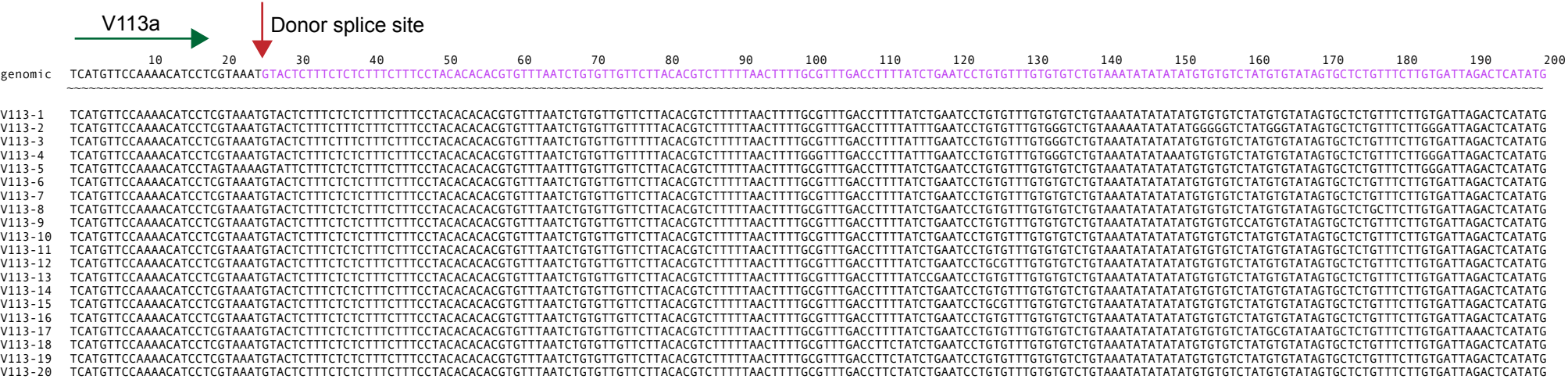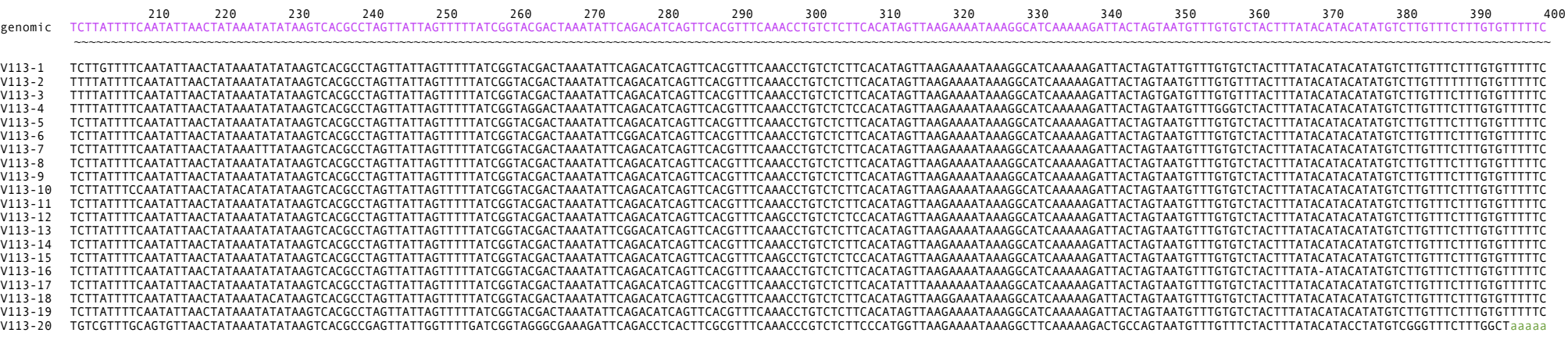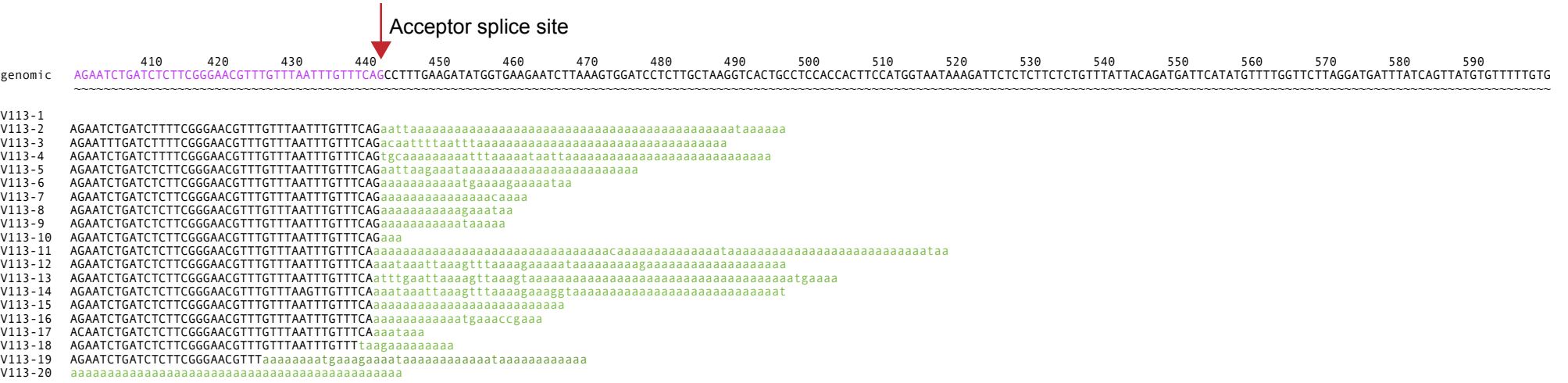

Supplement: Figure S10 — Unspliced transcripts from the At1g79270 locus are polyadenylated. Sequences of 3′ RACE PCR products obtained from hen2-4 samples. cDNA synthesis was initiated using oligo-dT as primer. 3′ RACE PCR was performed with V113a (green arrow) as forward primer, and the adapter sequence of the oligo-dT primer as a reverse primer. PCR products obtained from hen2-4 samples were cloned and sequenced. The genomic sequence is given above the line, with intronic sequence in purple. Red arrows mark donor and acceptor splice sites. Non-encoded nucleotides are in green. (PDF) [file pgen.1004564.s012.pdf]

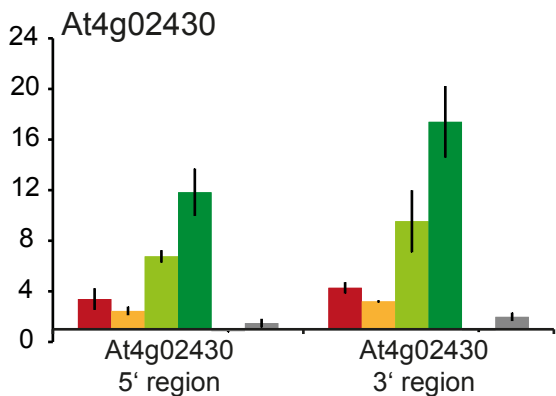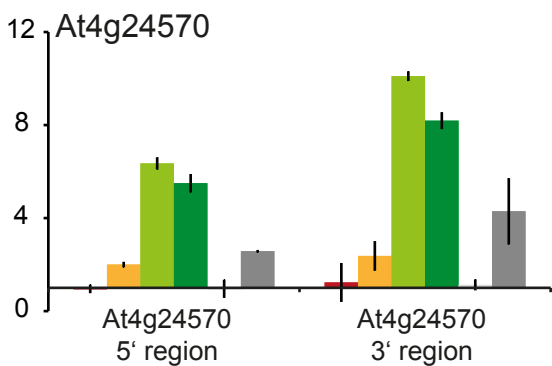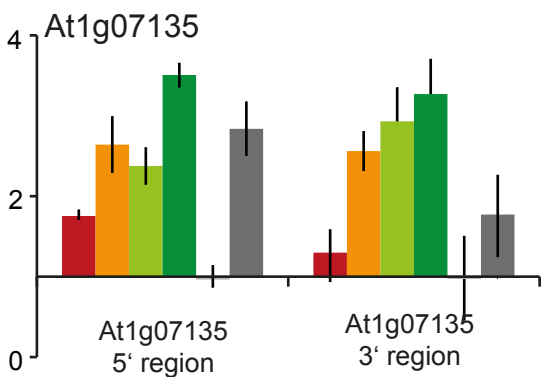

*mtr4-1* *hen2-2* *RRP41* ctrl  
*mtr4-2* *hen2-4* *RRP41* RNAi

Supplement: Figure S11 — Upregulation of mRNAs in hen2 mutants. Accumulation of mRNAs was tested by qRT-PCR using primer pairs located in 5′, central or 3′ regions of the annotated transcripts as indicated below each panel. mtr4-1 in red, mtr4-2 in orange, hen2-2 in light green, hen2-4 in dark green, RRP41 control in light grey, RRP41 RNAi in dark grey. Error bars = SD in three biological replicates. (PDF) [file pgen.1004564.s013.pdf]

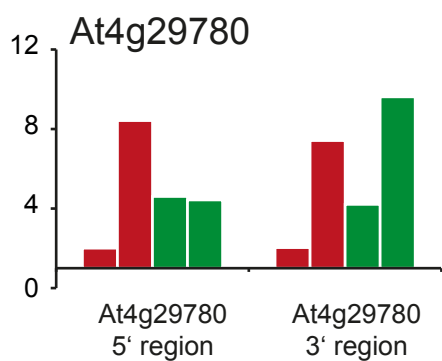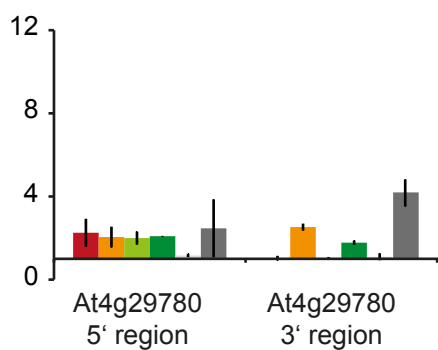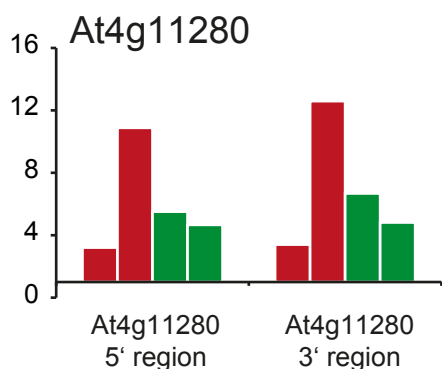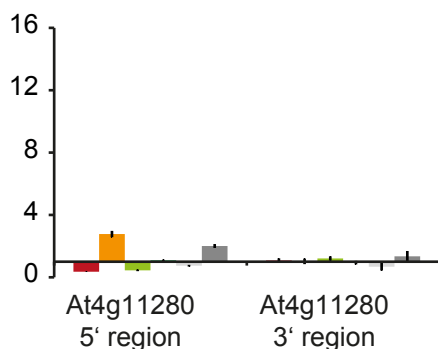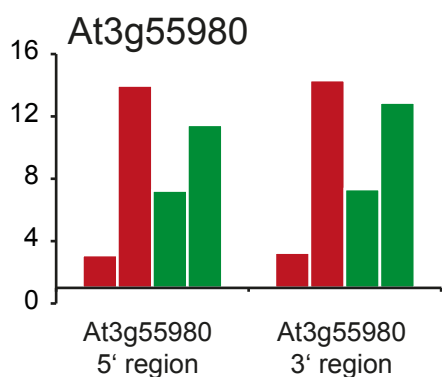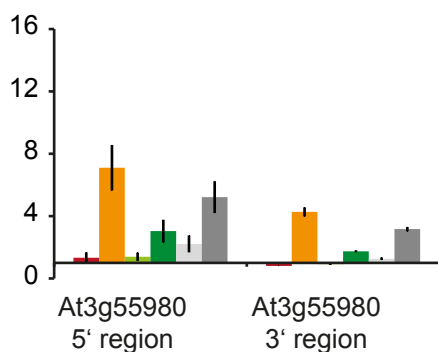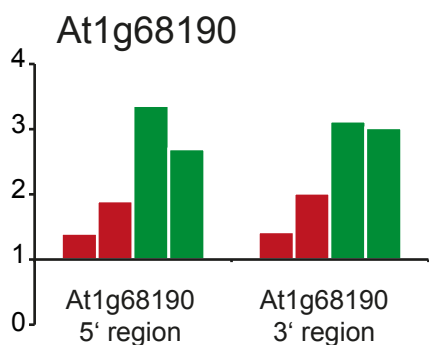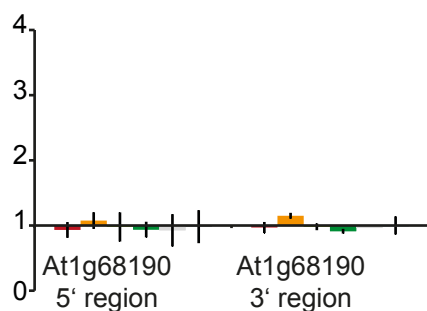

samples used for  
the tiling analysis

other samples grown  
in same culture conditions

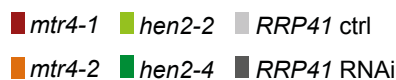

Supplement: Figure S12 — mRNAs are not systematically detected in all replicates. Accumulation of mRNAs was tested by qRT-PCR using primer pairs located in 5′ or 3′ regions of the annotated transcripts as indicated below each panel. Panels on the left show the qRT-PCR results for exactly the same samples that have been used for hybridisation to the tiling arrays. Panels on the right show the results obtained in 3 independent replicates grown in the same culture conditions. A possible explanation for the inconsistence between the replicates could be that mRNAs are not bona-fide substrates of exosome-mediated RNA degradation and might rather be upregulated due to indirect effects. Other types of transcripts such as short mRNA-derived regions, introns, unspliced transcripts or several types of non- coding RNAs are consistently observed in all replicates. mtr4-1 in red, mtr4-2 in orange, hen2-2 in light green, hen2-4 in dark green, RRP41 control in light grey, RRP41 RNAi in dark grey. Error bars = SD in three biological replicates. (PDF) [file pgen.1004564.s014.pdf]

WT    *mtr4-1*    *mtr4-2*    *hen2-2*    *hen2-4*    RRP41 Ctrl    RRP41 RNAi

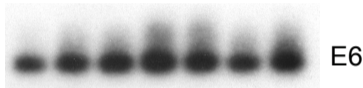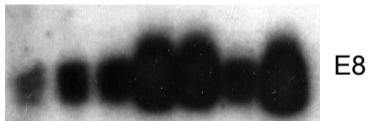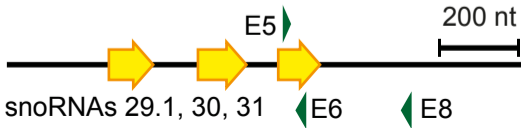

Supplement: Figure S13 — Loss of HEN2 or the exosome is associated with increased levels of polyadenylated snoRNA precursors. Oligo-dT primed cDNA was used for 3′ RACE- PCR, with primer E5 and the adapter sequence of the cDNA synthesis primer as forward and reverse primers, respectively. PCR products were separated on 2% agarose gels, transferred to Hybond XL membranes, and hybridized with radiolabeled probes E6 (mid panel) and E8 (lower panel). The diagram below illustrates location of primers and probes with respect to the snoRNA genes in this region (see also Fig. 9). (PDF) [file pgen.1004564.s015.pdf]

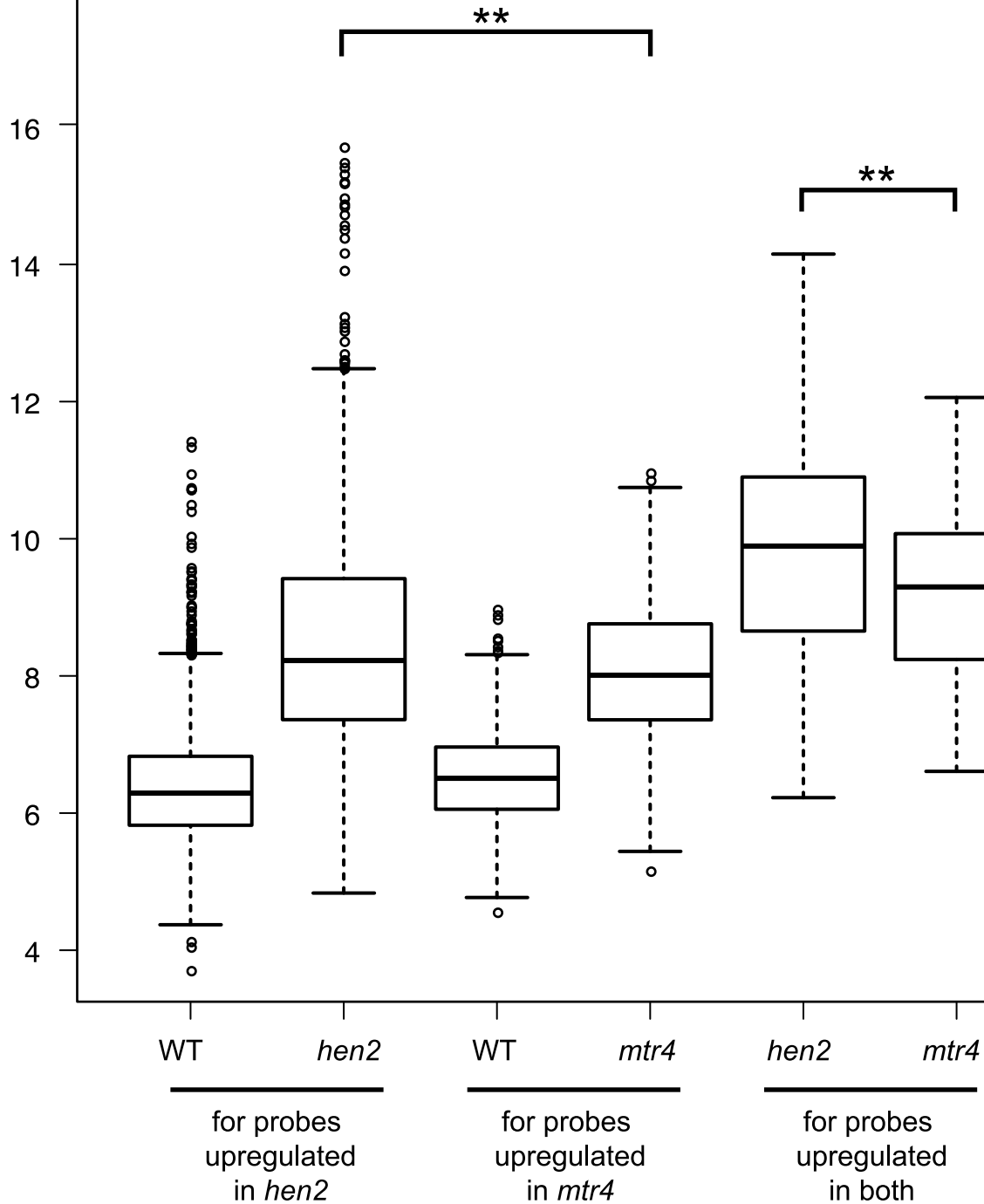

Supplement: Figure S15 — mtr4 mutants accumulate exosome substrates to lower levels than hen2 mutants. Boxplot showing averaged intensity values for the overexpressed probes identified in each comparison of the tiling array analysis. The first two rows show the intensity values in WT and hen2 samples for all probes overexpressed in both biological replicates of hen2. The third and fourth rows show the intensity values in WT and mtr4 samples for all probes overexpressed in both biological replicates of mtr4. The averaged intensities in mtr4 samples are significant lower than the averaged intensities in hen2 samples (p-value<1e -3). The fifth and sixth rows show the intensity values in hen2 and mtr4 for the common probes (overexpressed in both mutants). Again, the mean average intensity in mtr4 samples is lower than the mean average intensity in hen2 samples (p-value<1e -3). (PDF) [file pgen.1004564.s017.pdf]

AtMTR4-GFP

DAPI

Overlay

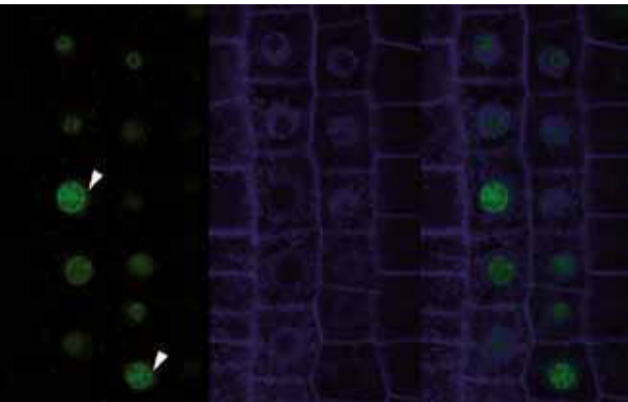

Supplement: Figure S17 — A fraction of AtMTR4-GFP can be detected in the nucleoplasm. Intracellular distribution of AtMTR4-GFP in root cells of a stable Arabidopsis transformant. The nucleoplasmic fraction of AtMTR4-GFP (white arrows) is more visible in individual transformants displaying relative weak transgene expression, which is not representative for the majority of the investigated plant lines. (PDF) [file pgen.1004564.s019.pdf]
