# Supplementary material for: The RNA Helicases AtMTR4 and HEN2 Target Specific Subsets of Nuclear Transcripts for Degradation by the Nuclear Exosome in Arabidopsis thaliana
Source: PLoS Genet. 2014 Aug 21;10(8):e1004564. doi: 10.1371/journal.pgen.1004564 (PMC4140647; doi:10.1371/journal.pgen.1004564)
Supplement: Figure S7 — Sequence alignment of human RBM7 and Arabidopsis At4g10110. (PDF) [file pgen.1004564.s009.pdf]

```
RBM7_HUMAN        MGAAAAEADRTLFVGNLETKVTEELLFELFHQAGPVIKVKIPKDKD-GKPKQFAFVNFKH
At4g10110_ARATH   ---MSGTSNCTVYIGNVDERVSDRVLYDIMIQAGRVIDLHIPRDKETDKPKGFAFAEYET
                      :. :: *:::**:: :*::.:*:::: *** **.::**:**:  *** ***.:::

RBM7_HUMAN        EVSVPYAMNLLNGIK-LYGRPIKIQFRSGSS-----------HAPQDVSLSYPQHHVGNS
At4g10110_ARATH   EEIADYAVKLFSGLVSLYNRTLKFAISGQDKLQSNSANSGHRARPQSL--AFEHSDR---
                  *  . **::*:.*:  ** * :*: : . ..             **.:  :: : .

RBM7_HUMAN        SPTSTSPSRYERTMDNMTSSAQIIQRSFSSPENFQRQAVMN--SALRQMSYGGKFGSSPL
At4g10110_ARATH   -------AAYHH-LER--FSSQLISPPSPLPLDYTQEPPPPGVSNGASLEYSRRVLGSAL
                         : *.: ::.   *:*:*.     * :: ::      *   .:.*. :. .* *

RBM7_HUMAN        DQSGFSPSVQSHSHSFNQSSSSQWRQGTPSSQRKVRMNSYPYLADRHYSREQRYTDHGSD
At4g10110_ARATH   D-------------SINHSR------------------------------PRRY------
                  *             *:*:*                                :**

RBM7_HUMAN        HHYRGKRDDFFYEDRNHDDWSHDYDNRRDSSRDGKWRSSRH
At4g10110_ARATH   -----------------------------------------
```
