# Supplementary material for: The RNA Helicases AtMTR4 and HEN2 Target Specific Subsets of Nuclear Transcripts for Degradation by the Nuclear Exosome in Arabidopsis thaliana
Source: PLoS Genet. 2014 Aug 21;10(8):e1004564. doi: 10.1371/journal.pgen.1004564 (PMC4140647; doi:10.1371/journal.pgen.1004564)
Supplement: Figure S8 — Supplemental information about selected known exosome substrates from Fig. 4. (DOCX) [file pgen.1004564.s010.docx]

Fig. S8: Supplemental information about selected known exosome targets (for Fig 4).

7SL RNA

Not correctly annotated in TAIR 10. In fact, the 7SL RNA is transcribed from the antisense region of AT4G02970. Accession number: X72228.1

The upregulation of 7SL-RNA detected by tiling array with exosome mutants can be seen on

<http://signal.salk.edu/cgi-bin/atta?GENE=At4g02970>

> 7SL RNA

GTCGAGCTAAGTAACATGAGCTTGTAACCCATGTGGGGACATTTAGATGGTGGAACACTGGTTCGGGTCCACGGGCCGGTTCTGTTGTTGGCATGTTTCTGGGCTGCCCAGTCCAAGCTGTGAGTAAGACGTGTGTGTCAAGCGAAGGCTTGGCTCAAACGGCTTCTAAAGTTGGAGGGTAATGCGTGAGGCTGGTTTCACAGAGCAGCGACTACTTCCCGCTTACAGCAGTGGACGGATCACAGTTTAGCGTCGCTCAGAACCACTATGGCCTGCTGGTCCGATCTCATATGAACCACCATTT

MRP RNA

Not annotated in TAIR 10. In fact, MRP RNA is transcribed from a locus that overlaps with AT3G63052. No Accession number. Reference for identifiation and sequence of MRP1 gene:

Kiss, T, Marshallsay C, and Filipowicz W. (1992) 7-2/MRP RNAs in Plant and Mammalian Cells: Association with Higher Order Structures in the Nucleolus. EMBO J 11, pp 3737–46.

The upregulation of MRP RNA detected by tiling array with exosome mutants can be seen on

<http://signal.salk.edu/cgi-bin/atta?GENE=At3g63052>

> MRP1

ACAATTGTCACTGGACGAAGTGAATGGGTCATATGGGCTTGTCCAAGTTCCGACCCAGGAAAGTCCCCGGGCCACTTATCCGCAGAGATGCGGCCTCGGTAACGAGAGAATCTTGCGGTGGAGAGATTCAAATTGCTGAGACGCGTGTGTGGAGCTTATGTGGTCTCTCCGCCGATGATATCATGGCCGTTCGACAGTTATTCACCTCTTCCTCTATGGACTAACTGAACGGGGCTTACGTTTCAATGACAAGCAACTTTT

dicistronic precursor of snoRNAs At3g58193 and At3g58196

The upregulation of this region detected by tiling array with exosome mutants can be seen on

<http://signal.salk.edu/cgi-bin/atta?GENE=At3g58193>

In our experiment, the forward qRT-PCR primer is located in At3g58193, the reverse primer is located in At3g58196. Therefore, the qPCR amplicon queries a dicistronic precursor transcript.

Intergenic repeats

Repeat region located on Chromosome 5 (between At5g15530 and At5g15540)

The upregulation of this region detected by tiling array with exosome mutants can be seen on

<http://signal.salk.edu/cgi-bin/atta?JOB=ALIGN&QUERY=&CHROMOSOME=chr5&COORS=C/5045742-5045840>

Locus At2g18440

Contains two non-coding RNAs annotated as GUT15 (for Gene with Unstable Transcripts 15)

The upregulation of this region detected by tiling array with exosome mutants can be seen on

<http://signal.salk.edu/cgi-bin/atta?JOB=ALIGN&QUERY=&CHROMOSOME=chr2&COORS=C/7995662-7995802>

Pseudogene At1g79245

The upregulation of this region detected by tiling array with exosome mutants can be seen on

<http://signal.salk.edu/cgi-bin/atta?GENE=At1g79245>
