# Supplementary material for: The RNA Helicases AtMTR4 and HEN2 Target Specific Subsets of Nuclear Transcripts for Degradation by the Nuclear Exosome in Arabidopsis thaliana
Source: PLoS Genet. 2014 Aug 21;10(8):e1004564. doi: 10.1371/journal.pgen.1004564 (PMC4140647; doi:10.1371/journal.pgen.1004564)
Supplement: Figure S14 — Polyadenylated transcripts partially antisense to AT5G44306. Sequences were amplified by 3′ RACE from oligo-dT primed cDNA from the indicated samples using a primer (indicated by the purple arrow) situated antisense to the 5′ region of AT5G44306. Non-encoded nucleotides are in green. (PDF) [file pgen.1004564.s016.pdf]

genomic (reverse complement)

CCAATAAGAGTATCTCATGGGAGCTGAATGAACAGCGGATAAGTTTCCTCTCCCAGATTTGGATTTCTATAAGATACTAATCTGGACGTGGCTCTTCCCTGATCTTGAGAGTTGACCCACGGTCGAAACAGGATTTGGTCTAAATGTCTATCCCAGAGATCATGCATTTGGGTTAGAGAATTGAGGTAATATTAATTACTTTCTTTTCGGTTTTATATATGATAATTGATAAGA

hen2: 28 out of 32 clones corresponded to target

CCAATAAGAGTATCTCATGGGAGCTGAATGAACAGCGGATAAGTTTCCTCTCCCAGATTTGGATTTCTATAAGATACTAATCTGGACGTGGCTCTTCCCTGATCTTGAGAGTTGACCCACGGTCGAAACAGGATTTGGTCTAAATGTCTATCCCAGAGATCATGCATTTGGGTTAGAGAATTGAGGTAATATTAATTACTTTCTTTTaaaaaaaaaaaaaaaaaaataaa
CCAATAAGAGTATCTCATGGGAGCTGAATGAACAGCGGATAAGTTTCCTCTCCCAGATTTGGATTTCTATAAGATACTAATCTGGACGTGGCTCTTCCCTGATCTTGAGAGTTGACCCACGGTCGAAACAGGATTTGGTCTAAATGTCTATCCCAGAGATCATGCATTTGGGTTAGAGAATTGAGGTAATATTAATTGCTTTCTTTTaaaaaaaaaaaaaaaa
CCAATAAGAGTATCTCATGGGAGCTGAATGAACAGCGGATAAGTTTCCTCTCCCAGATTTGGATTTCTATAAGATACTAATCTGGACGTGGCTCTTCCCTGATCTTGAGAGTTGACCCACGGTCGAAACAGGATTTGGTCTAAATGTCTATCCCAGAGATCATGCATTTGGGTTAGAGAATTGAGGTAATATTAATTACTTTCTTaaaaaaaaaaaaaaaaaaaaa
CCAATAAGAGTATCTCATGGGAGGTGAATGAACAGCGGATAAGTTTCCTCTCCCAGATTTGGATTTCTATAAGATATTAATCTGGACGTGGCTCTTCCCTGATCTTGAGAGTTGACCCACGGTGGAAACAGGATTTGGTTTAAATGTTTATCCCAGAGATCATGCATTTGGGTTAGAGAATTGAGGTAATATTAATTACTTTTTTaaaaaaaaaaaaaaaaaaaaaaaaaaaaaaaaaaaaaaaaa
CCAATAAGAGTATCTCATGGGAGGTGAATGAACAGCGGATAAGTTTCCTCTCCCAGATTTGGATTTCTATAAGATATTAATCTGGACGTGGCTCTTCCCTGATCTTGAGAGTTGACCCACGGTGGAAACAGGATTTGGTTTAAATGTCTATCCCAGAGATCATGCATTTGGGTTAGAGAATTGAGGTAATATTAATTACTTTTTTaaaagaagaaaaaaaaaaaaaaaaa
CCAATAAGAGTATCTCATGGGAGGTGAATGAACAGCGGATAAGTTTCCTCTCCCAGATTTGGATTTCTATAAGATATTAATCTGGACGTGGCTCTTCCCTGATCTTGAGAGTTGACCCACGGTCGAAACAGGATTTGGTCTAAATGTCTATCCCAGAGATCATGCATTTGGGTTAGAGAATTGAGGTAATATTAATTACTTTCTTaaaaaaaaaaaaa
CCAATAAGAGTATCTCATGGGAGGTGAATGAACAGCGGATAAGTTTCCTCTCCCAGATTTGGATTTCTATAAGATATTAATCTGGACGTGGCTCTTCCCTGATCTTGAGAGTTGACCCACGGTGGAAACAGGATTTGGTCTAAATGTCTATCCCAGAGATCATGCATTTGGGTTAGAGAATTGAGGTAATATTAATTACTTTCTTaaaagaagaaaaaaaaaaaaaaaa
CCAATAAGAGTATTTCATGGGAGGTGAATGAACAGCGGATAAGTTTCCTTTCCCCGATTTGGATTTTTATAAGATACTAATTTGGGCGGGGCTTTTCCCTGATCTTGAGAGTTGACCCCCGGTCGAAACAGGATTTGGTTTAAATGTTTATCCCAGAGATCATGCATTTGGGTTAGAGAATTGAGGTAATATTAATTACTTTTTTaaagaaaaataaagataaaaaaaaaaaaaaaaaaaaaaaaaaaaaaagaaaaaaaaaaaaaaa
CCAATAAGAGTATCTCTTGGGGGATGAATGAACAGAGGATAAGTTTCCTCTCCCCGATTTGGATTTTTATAAGATATTAATCTGGACGTGGCTCTTCCCTGATCTTGAGAGTTGACCCACGGTGGAAACAGGATTTGGTGTAAAAGTGTATCCCCGAGATCATGCATTTGGGTTAGAGAAATGAGGTAATATTAATTACTTTCTTaaaaaaaaaaaaaaaaaaaaa
CCAATAAGAGTATCTCATGGGAGCTGAATGAACAGCGGATAAGTTTCCTCTCCCAGATTTGGATTTCTATAAGATACTAATCTGGACGTGGCTCTTCCCTGATCTTGAGAGTTGACCCACGGTCGAAACAGGATTTGGTCTAAATGTCTATCCCAGAGATCATGCATTTGGGTTAGAGAATTGAGGTAATATTAATTACTTTCTataaaaaaagaaaaagaaaaaaaaggaaaaaaaaaaa
CCAATAAGAGTATCTCATGGGAGCTGAATGAACAGCGGATAAGTTTCCTCTCCCAGATTTGGATTTCTATAAGATACTAATCTGGACGTGGCTCTTCCCTGATCTTGAGAGTTGACCCACGGTCGAAACAGGATTTGGTCTAAATGTCTATCCCAGAGATCATGCATTTGGGTTAGAGAATTGAGGTAATATTAATTACTTTCTaaaaaaaaagaaaaaaaaaaaa
CCAATAAGAGTATTTCATGGGAGCTGAATGAACAGCGGATAAGTTTCCTCTCCCAGATTTGGATTTCTATAAGATACTAATCTGGACGTGGCTCTTCCCTGATCTTGAGAGTTGACCCACGGTCGAAACAGGATTTGGTTTAAATGTCTATCCCAGAGATCATGCATTTGGGTTAGAGAATTGAGGTAATATTAATTACTTaaaaaaaaaaaaaaaaaaaaaaaa
CCAATAAGAGTATCTCATGGGAGCTGAATGAACAGCGGATAAGTTTCCTCTCCCAGATTTGGATTTCTATAAGATACTAATCTGGACGTGGCTCTTCCCTGATCTTGAGAGTTGACCCACGGTCGAAACAGGATTTGGTCTAAATGTCTATTTCAGAGATCATGCATTTGGGTTAGAGAATTGAGGTAATATTAATTACTaagaaaaaaaaaaaaaaaaa
CCAATAAGAGTATTTCATGGGAGCTGAATGAACAGCGGATAAGTTTCCTCTCCCCGATTTGGATTTCTATAAGATACTAATCTGGACGTGGCTTTTCCCTGATCTTGAGAGTTGACCCACGGTCGAAACAGGATTTGGTTTAAATGTCTATCCCAGAGATCATGCATTTGGGTTAGAGAATTGAGGTAATATTAATTAaaaaggaagaagaaaaaaaaaaaaa
CCAATAAGAGTATTTCATGGGAGCTGAATGAACAGCGGATAAGTTTCCTCTCCCAGATTTGGATTTCTATAAGATACTAATCTGGACGTGGCTCTTCCCTGATCTTGAGAGTTGACCCACGGTCGAAACAGGATTTGGTCTAAATGTCTATCCCAGAGATCATGCATTTGGGTTAGAGAATTGAGGTAATATTAATaaaaaaaaaaaaaaaaagaaaaaagaaagaaaaaaaaaaaaaa
CCAATAAGAGTATCTCATGGGAGCTGAATGAACAGCGGATAAGTTTCCTCTCCCAGATTTGGATTTCTATAAGATACTAATCTGGACGTGGCTCTTCCCTGATCTTGAGAGTTGACCCACGGTCGAAACAGGATTTGGTCTAAATGTCTATCCCAGAGATCATGCATTTGGGTTAGAGAATTGAGGTAACATTAAaaaaaaaagaaaaaaaaaaa
CCAATAAGAGTATCTCATGGGAGCTGAATGAACAGCGGATAAGTTTCCTCTCCCAGATTTGGATTTCTATAAGATACTAATCTGGACGTGGCTCTTCCCTGATCTTGAGAGTTGACCCACGGTCGAAACAGGATTTGGTCTAAATGTCTATCCCAGAGATCATGCATTaaagagaaaaaagaagaaaaaaaaaaaaaaa
CCAATAAGAGTATCTCATGGGAGCTGAATGAACAGCGGATAAGTTTCCTCTCCCAGATTTGGATTTCTATAAGATACTAATCTGGACGTGGCTCTTCCCTGATCTTGAGAGTTGACCCACGGTCGAAACAGGATTTGGTCTAAATGTCTATCCCAGAGATCATGCATaaaaaaaaaaaaaaaaaaa
CCAATAAGAGTATCTCATGGGAGCTGAATGAACAGCGGATAAGTTTCCTCTCCCAGATTTGGATTTCTATAAGATACTAATCTGGACGTGGCTCTTCCCTGATCTTGAGAGTTGACCCACGGTCGAAACAGGATTTGGTCTAAATGTCTATCCCAGAGATCATGCAaaaaagaaaaaaaaaaaaa
CCAATAAGAGTATCTCATGGGAGCTGAATGAACAGCGGATAAGTTTCCTCTCCCAGATTTGGATTTCTATAAGATACTAATCTGGACGTGGCTCTTCCCTGATCTTGAGAGTTGACCCACGGTCGAAACAGGATTTGGTCTAAATGTCTATCCCAGAGATCATGaaaaaaaaaaaaa
CCAATAAGAGTATCTCATGGGAGGTGAATGAACAGCGGATAAGTTTCCTCTCCCAGATTTGGATTTCTATAAGATATTAATCTGGACGTGGCTCTTCCCTGATCTTGAGAGTTGACCCCCGGTCGAAACAGGATTTGGTTTAAATGTCTATCCCAaaaaagaaaaataaaaaaa
CCAATAAGAGTATCTCATGGGAGCTGAATGAACAGCGGATAAGTTTCCTCTCCCAGATTTGGATTTCTATAAGATACTAGTCTGGACGTGGCTCTTCCCTGATCTTGAGAGTTGACCCACGGTCGAAACAGGATTTGGaaaaaagaaaaaaaaagagaaaaaaaaaaaaaaaaaaaaaaa
CCAATAAGAGTATCTCATGGGAGCTGAATGAACAGCGGATAAGTTTCCTCTCCCAGATTTGGATTTCTATAAGATACTAATCTGGACGTGGCTCTTCCCTGATCTTGAGAGTTGACCCACGGTCGAAACAGGATTTGGgaaaaaaaaaaaaaaaa
CCAATAAGAGTATCTCATGGGAGGTGAATGAACAGCGGATAAGTTTCCTCTCCCAGATTTGGATTTCTATAAGATATTAATCTGGACGTGGCTCTTCCCTGATCTTGAGAGTTGACCCACGGTCGAAACAGGATTTcaaaaaaaaaaaaaaaaaaaaaaaaaaa
CCAATAAGAGTATCTCATGGGAGCTGAATGAACAGCGGATAAGTTTCCTCTCCCAGATTTGGATTTCTATAAGATACTAATCTGGACGTGGCTCTTCCCTGATCTTGAGAGaaaaaaaaaaaa
CAATAAGAGTATCTCATGGGAGGGGAATGAACAGCGGATAAGTTTCTTCTCCCAGATTTGGATTTCTATAAGATAaaaaaaaaaaaaaaataaagaa
CCAATAAGAGTATTTCATGGGAGCTGAATGAACAGCGGATAAGTTTCCTCTCCCAGATTTGGATTTCaaaagaaaaaaaaaaaaa
CCAATAAGAGTATCTCATGGGAGCTGAATGAACAGCGGATAAGTTTCCTCTCCCAGATTTGGATTTaaaagaagaaaaaaaaaaaaaa

mtr4: 3 out of 32 clones corresponded to target

CCAATAAGAGTATCTCATGGGAGCTGAATGAACAGCGGATAAGTTTCCTCTCCCAGATTTGGATTTCTaaaaaaaaaaaaaaaaa
CCAATAAGAGTATCTCATGGGAGCTGAATGAACAGCGGATAAGTTTCCTCTCCCAGATTTGGATTTaaaaaaaaaaaaaaaaaaaaa
CCAATAAGAGTATCTCATGGGAGCTGAATGAACAGCGGTTAAGTTTCCTTTCCCAGATTTAGATTTaaaaaaaaaaaaaaaaaaaaaaaaaaaaaacaaaaaaaaaaaaa

WT: 0 out of 32 clones corresponded to target
