# Supplementary material for: The RNA Helicases AtMTR4 and HEN2 Target Specific Subsets of Nuclear Transcripts for Degradation by the Nuclear Exosome in Arabidopsis thaliana
Source: PLoS Genet. 2014 Aug 21;10(8):e1004564. doi: 10.1371/journal.pgen.1004564 (PMC4140647; doi:10.1371/journal.pgen.1004564)
Supplement: Figure S16 — The microarray analysis probably underestimates the contribution of HEN2 to nuclear RNA surveillance. A diagram of the genomic locus indicated by the respective AGI number is shown at the top of each panel. Annotated mRNA genes are represented as arrows with dark blue boxes for the CDS, light blue boxes for 3′ and 5′ UTRs, and a light blue line for introns. Red bars above the diagram represent probes detected in the microarray analysis. Green arrows above or below the diagram depict the location of qRT-PCR primers. The corresponding qRT-PCR results for each primer pair are given as fold-change relative to WT in the histograms below each diagram. mtr4-1 in red, mtr4-2 in orange, hen2-2 in light green, hen2-4 in dark green, RRP41 control in light grey, RRP41 RNAi in dark grey. Error bars = SD in three biological replicates. Upper panel: The upregulation of two stretches in the 5′ region of At1g20100 in RRP41 RNAi lines (indicated by the red double arrows) was detected in a previous tiling microarray study [5]. Our microarray array detected only a portion of this region (indicated by the red bars above the diagram). However, we could confirm the upregulation of the uppermost 600 kb of At1g20100 in both hen2 alleles by qRT-PCR. Middle panel: A portion of the fifth intron of At5g27720 was previously identified as a target of the exosome core complex [5] (indicated by the red double arrows). In our microarray analysis, only one probe in this region was declared statistically significant. Since we considered only regions with at least two consecutive probes, this region was omitted from the data interpretation. Nevertheless, qRT-PCR data show that a portion of the fifth intron is upregulated in hen2 mutants, while levels of pre-mRNA or mature mRNAs are similar to WT. The presence of polyadenylated transcripts corresponding either to the entire intron or to shorter degradation intermediates in hen2 and RRP41 RNAi samples was further confirmed by cloning of 3′ RACE products [file pgen.1004564.s018.pdf]

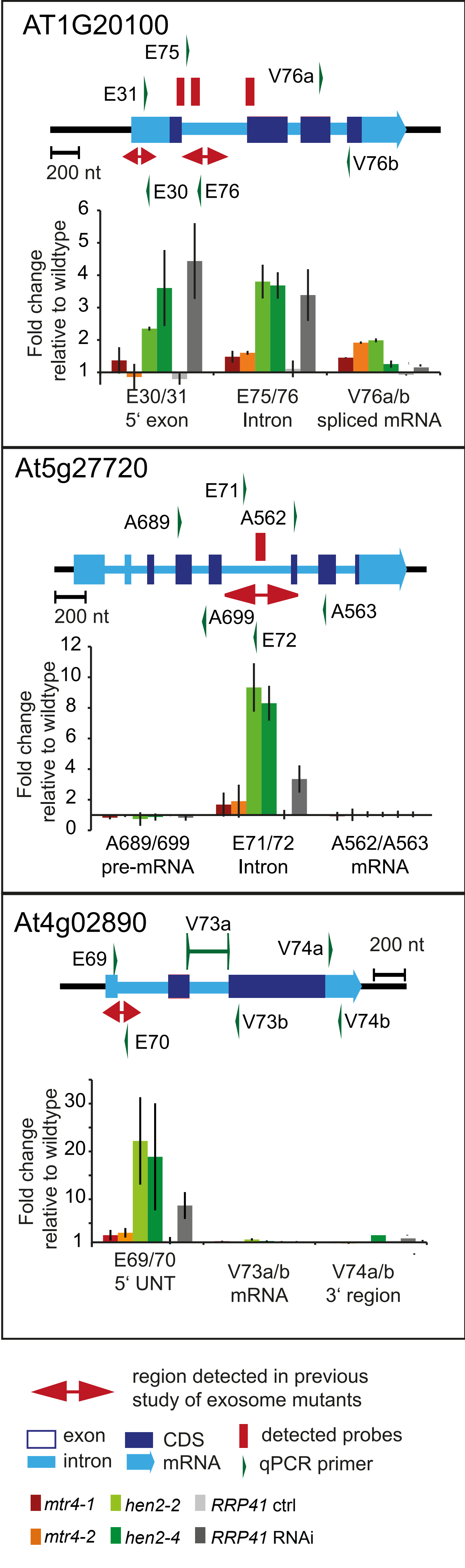

AT1G20100
E75
V76a
E31
200 nt
V76b
E30
E76
Fold change relative to wildtype
E30/31
5‘ exon
E75/76
Intron
V76a/b
spliced mRNA
At5g27720
E71
A689
A562
200 nt
A699
A563
E72
A689/699
pre-mRNA
E71/72
Intron
A562/A563
mRNA
At4g02890
V73a
V74a
200 nt
E69
V73b
V74b
E70
E69/70
5‘ UNT
V73a/b
mRNA
V74a/b
3‘ region
region detected in previous study of exosome mutants
exon
intron
CDS
mRNA
detected probes
qPCR primer
mtr4-1
hen2-2
RRP41 ctrl
mtr4-2
hen2-4
RRP41 RNAi
